# A global optimization paradigm based on change of measures

**DOI:** 10.1098/rsos.150123

**Published:** 2015-07-01

**Authors:** Saikat Sarkar, Debasish Roy, Ram Mohan Vasu

**Affiliations:** 1 Computational Mechanics Laboratory, Department of Civil Engineering, Indian Institute of Science, Bangalore 560012, India; 2 Department of Instrumentation and Applied PhysicsIndian Institute of Science, Bangalore 560012, India

**Keywords:** local and global extremizations, martingale problem, random perturbations, particle swarm optimization, differential evolution, covariance matrix adaptation evolution strategy

## Abstract

A global optimization framework, COMBEO (Change Of Measure Based Evolutionary Optimization), is proposed. An important aspect in the development is a set of derivative-free additive directional terms, obtainable through a change of measures *en route* to the imposition of any stipulated conditions aimed at driving the realized design variables (particles) to the global optimum. The generalized setting offered by the new approach also enables several basic ideas, used with other global search methods such as the particle swarm or the differential evolution, to be rationally incorporated in the proposed set-up via a change of measures. The global search may be further aided by imparting to the directional update terms additional layers of random perturbations such as ‘scrambling’ and ‘selection’. Depending on the precise choice of the optimality conditions and the extent of random perturbation, the search can be readily rendered either greedy or more exploratory. As numerically demonstrated, the new proposal appears to provide for a more rational, more accurate and, in some cases, a faster alternative to many available evolutionary optimization schemes.

## 1. Introduction

Potential applications of global optimization are ubiquitous across a large spectrum of problems in science and engineering, from very-large-scale integration circuit design, to optimization of transportation routes to determination of protein structures. An essential aspect of the solution to any such problem is the extremization of an objective functional, possibly subject to a prescribed set of constraints. The solution is deemed to have been achieved if the global extremum of the objective functional is reached. For many practical applications, the objective functional could be non-convex, non-separable and even non-smooth. This precludes gradient-based local optimization approaches to treat these problems, an aspect that has driven extensive research in devising global optimization strategies, many of which employ stochastic (i.e. random evolutionary) search of heuristic or meta-heuristic origin [1]. Indeed, finding the global optimum within a search space of the parameters or design variables is much more challenging than arriving at a local extremum. In the absence of generic directional information (equivalent to the Gateaux or directional derivative in the search for a local extremum of a sufficiently smooth cost functional), a direct search seems to be the only option left. Some of the notable schemes in this genre include variants of the genetic algorithm (GA) [2], differential evolution (DE) [3], particle swarm optimization (PSO) [4], ant colony optimization [5] and covariance matrix adaptation evolution strategy (CMA-ES) [6]. In a search for the global extremum, stochastic schemes typically score over their gradient-based deterministic counterparts [7,8], even for cases involving sufficiently smooth cost functionals with well-defined directional derivatives. Nevertheless, thanks to the proper directional information guiding the update, gradient-based methods possess the benefit of faster convergence to the nearest extremum, as long as the objective functional is sufficiently smooth. To the authors' knowledge, the existing evolutionary global optimization schemes do not offer the benefit of well-founded directional information. Most evolutionary schemes depend on a random (diffusion-type) scatter applied to the available candidate solutions or particles and some criteria for selecting the new particles. Unfortunately, a ‘greedy’ approach for the global optimum (e.g. selecting particles of higher ‘fitness’ with higher probability), though tempting from the perspective of faster convergence, faces the hazard of getting trapped in local extrema. As a fix to a premature and possibly wrong convergence, many evolutionarily global search schemes adopt safeguards. Despite the wide acceptability of a few evolutionary methods of the heuristic/meta-heuristic origin [9], the underlying justification is often based on sociological or biological metaphors [1–6,10] that are hardly founded on a sound probabilistic basis even though a random search forms a key ingredient of the algorithm. In this context, some interesting comments on the facile usage of natural metaphors in the development of many global optimization algorithms, without a serious engagement with scientific ratiocination, may be found in [10]. Be that as it may, the popular adoption of these schemes is not only owing to the algorithmic simplicity, but mainly because of their effectiveness in treating many non-deterministic polynomial-time hard optimization problems, which may be contrasted with a relatively poorer performance of some of the well-grounded stochastic schemes, e.g. simulated annealing [11], stochastic tunnelling [12], etc. Whether a slow convergence to the global optimum, not infrequently encountered with some of the popular evolutionary schemes, could be fixed by reorienting them with an alternative stochastic framework, however, remains a moot point, which is addressed in this work to an extent. Another related question is regarding the precise number of parameters in the algorithm that the end-user must tune for an appropriate *exploration–exploitation trade-off* [13,14], a phrase used to indicate the relative balance of random scatter of the particles vis-a-vis their selection based on the ‘fitness’. Admittedly, the notion of random evolution, built upon Monte Carlo (MC) sampling of the particles, efficiently explores the search space, though at the cost of possibly slower convergence [15] in contrast to a gradient-based method. For better exploration, many such schemes, e.g. the GA, adopt steps like ‘crossover’, ‘mutation’, ‘selection’, etc. While ‘crossover’ and ‘mutation’ impart variations in the particles, by the ‘selection’ step each particle is assigned a ‘weight’ or fitness value (a measure of importance) determined as the ratio of the realized value of the objective functional to the available maximum of the same. In the selection step, the fitness values, used to update the particles via selection of the best-fit individuals for subsequent crossover and mutation, may be considered functionally analogous to the derivatives used in gradient-based approaches. Analogy may also be drawn between the fitness values and the likelihood ratios commonly employed in nonlinear stochastic filtering, e.g. the family of sequential importance sampling filters [16]. Even though a weight-based approach reduces a measure of misfit between the available best and the rest within a finite ensemble of particles, they bring with them the curse of degeneracy, wherein all particles but one tend to receive zero weights as the iterations progress [17]. This problem, also referred to as ‘particle collapse’ in the stochastic filtering parlance, can be arrested, in principle, by exponentially increasing the ensemble size (number of particles), a requirement that can hardly be met in practice [18]. Evolutionary schemes that replace the weight-based multiplicative approach by adopting additive updates for the particles, often succeed in eliminating particle degeneracy. Evolutionary schemes like DE, PSO, etc., that are known to perform better than GA, use such additive particle update strategies without adversely affecting the ‘craziness’ or randomness in the evolution. On reaching the optimum, the additive correction shrinks to zero. Unfortunately, none of these methods obtain the additive term, which may be looked upon as a stochastic equivalent to the derivative-based directional information, in a rigorous or optimal manner—a fact that is perhaps responsible for a painstakingly large number of functional evaluations in some cases.

In framing a rigorous stochastic basis leading to directional updates of the additive type, several possible schemes are suggested in this work. First, it is shown that the problem of optimization may be generically posed as a *martingale problem* [19,20], which must however be randomly perturbed to facilitate the global search. For instance, one may perform a greedy search for an extremum of an objective functional by solving a martingale problem, which is in the form of an integro-differential equation herein referred to as the extremal equation. In line with the Freidlin–Wentzell theory [21], the martingale problem may further be perturbed randomly so as to enable the greedy scheme to converge to the global extremum as the strength of the perturbation vanishes asymptotically. The solution through this approach, which is viewed as a stochastic process parametered via the iterations, depends crucially on an error or ‘innovation’ function and is deemed to have been realized when the innovation function, interpreted as a stochastic process, is driven to a zero-mean noise or martingale [19]. The martingale structure of the innovation essentially implies that the mean of the computed cost functional in future iterations remains constant and invariant to zero-mean random perturbations of the argument vector (the design variables). The argument vector should then correspond to an extremum. In the evolutionary random set-up, both the cost functional and its argument vector are treated as stochastic diffusion processes even though the original extremization problem is deterministically posed. Realizing a zero-mean martingale structure for the innovation requires a change of measures that in turn leads to additive gain-type updates on the particles. The gain coefficient matrix, which is a replacement for and generalization over the Frechet derivative of a smooth cost functional, provides an efficacious directional search without requiring the functional to be differentiable. Additionally, in order to facilitate the global search, a general random perturbation strategy (using steps such as ‘scrambling’, ‘relaxation’ and ‘selection’) is adopted to insure against a possible trapping of particles in local extrema.

An important feature of the present approach is the flexibility with which the innovation process may be designed in order to meet a set of conflicting demands *en route* the detection of the global extremum. The efficiency of the global search basically relies upon the ability of the algorithm to explore the search space while preserving some directionality that helps to quickly resolve the nearest extremum. The development of the proposed set-up, referred to as COMBEO (**C**hange **O**f **M**easure **B**ased **E**volutionary **O**ptimization), recognizes the near impossibility of a specific optimization scheme performing uniformly well across a large class of problems. In this context, one may refer to variants of the famous ‘no free lunch theorem’, where it is proved that the average performance of any two algorithms across all possible problems is identical [22]. Accommodation of this fact had earlier led to a proposal of an evolutionary scheme that simultaneously ran different optimization methods for a given problem with some communications built among the updates by the different methods [23]. A major related contribution of this work is to bring the basic ideas for global search used in a few well-known optimization schemes under a single unified framework propped up by a sound probabilistic basis. In a way explained later, the ideas (or their possible generalizations) behind some of the existing optimization methods may sometimes be readily incorporated in the present setting by just tweaking the innovation process.

The rest of the paper is organized as follows. In §2, the problem of finding an extremum of an objective functional is posed as a martingale problem represented through an integro-differential equation, whose solution only realizes a local optimum. The integro-differential equation is discretized and weakly solved within an MC setting so as to circumvent its inherent circularity. Section 3 discusses several random perturbation schemes to arrive at the global optimum efficiently without getting stuck in local traps. By combining these tools in different ways, a few pseudo-codes are presented in §4. In §5, the performance of COMBEO is compared with DE, PSO and CMA-ES in the context of several benchmark problems. In the same section, the new optimization approach is also applied for the quantitative recovery of boundary layer density variation in a high-speed flow given the light travel time data from an experiment. Finally, the conclusions of this study are drawn in §6.

## 2. Search for an extremum through a martingale problem

Here, it is demonstrated that functional extremization, subject to a generic set of equality constraints, can be posed as a martingale problem upon a proper characterization of the design variables within a probabilistic setting. However, before elaborating on this, a few fundamental features of the new evolutionary approach are listed below:

(i) at a given iterate, the solution is a random variable (taking values in the search space);
(ii) hence the solution process along the iteration axis may be considered a stochastic process and the iterations must be so designed that the optimal solution is asymptotically approached by the mean (i.e. the first moment) of the solution process; and
(iii) since upon convergence the mean should be iteration-invariant, random fluctuations about the converged mean must assume the structure of a zero-mean stochastic process (along the iteration axis), thereby allowing us to identify the fluctuations as a noise process, e.g. a Brownian motion, or, more generally, a zero-mean martingale.

In posing the global optimization problem within a stochastic framework, a complete probability space (Ω,F,P) [19] is considered, within which the solution to the optimization problem must exist as an F-measurable random variable. Here *Ω*, known as the population set (sample space), necessarily includes all possible candidate solutions from which a finite set of randomly chosen realizations or particles is evolved along the iteration axis *τ*. The introduction of *τ* as a positive monotonically increasing function on R is required to qualify the evolution of the solution as a continuously parametered stochastic process. A necessary aspect of the evolution is a random initial scatter imparted to the particles so as to search the sample space for solutions that extremize the cost functional while satisfying the posed constraints, if any. Since the extremal values, global or otherwise, of the objective functional may not be known *a priori*, the particles are updated iteratively (i.e. along *τ*) by conditioning on the evolution history of a so-called extremal cost process based on the available particles. The extremal cost process is defined as a stochastic process such that, for any *ξ*∈[0,*τ*], its mean is given by the available best objective functional based on its evaluations across the particles at the iteration step denoted by *ξ*. Now denoting by $N_{\tau }:={\left\{G_{\xi }\right\}}_{\xi \in [0,\tau ]}$ the filtration (i.e. statistical information pertaining to the evolution history) containing the increasing family of sub *σ*-algebras generated by the extremal cost process, the aim is to determine the update by conditioning the guessed or predicted solution on $N_{\tau }$. The guessed solution could be a Brownian motion or a random walk along *τ*. In case there are equality constraints, $N_{\tau }$ also contains history for the best realized constraint until *τ*. For a multivariate multimodal nonlinear objective functional $f(\text{x}):R^{n}\mapsto R$ in $\text{x}={\left\{x^{j}\right\}}_{j=1}^{n}\in R^{n}$, a point x* needs to be found such that $f({\text{x}}^{*})\leq f(\text{x}),\;\forall \;\text{x}\in R^{n}$. Since the design variable **x** is evolved in *τ* as a stochastic process, it is parametrized as **x**_*τ*_:=**x**(*τ*). It may be noted that there may not be any inherent physical dynamics in its evolution, and in such cases **x**_*τ*_ may be evolved as a zero-mean Brownian motion or random walk in *Ω* (even though, as will be shown later, this step is not absolutely necessary). Since only a finite number of iterations can be performed in practice, *τ* is discretized as *τ*_0_<*τ*_1_<⋯<*τ*_*M*_ so that **x**_*τ*_ is evolved over every *τ* increment. Thus, for the *i*th iteration with *τ*∈(*τ*_*i*−1_,*τ*_*i*_], **x**_*τ*_ may be thought of as being governed by the following stochastic differential equation (SDE):

2.1 $$d{\text{x}}_{\tau }=d\xi_{\tau }.$$

Here ***ξ***_*τ*_ is a zero-mean vector Brownian process with the covariance matrix $\text{g}{\text{g}}^{T}\in R^{n\times n}$, where $\text{g}\in R^{n\times n}$ is the noise intensity matrix with its (*j*,*k*)th element denoted as *g*^*jk*^. The discrete *τ*-marching map for equation (2.1) has the form:

2.2 $${\text{x}}_{i}={\text{x}}_{i-1}+\Delta \xi_{i},\;\Delta \xi_{i}:=\xi_{i}-\xi_{i-1},$$

where (⋅)_*i*_ stands for (⋅)_*τ*_*i*__. Let the extremal cost process, generating the filtration $N_{\tau }$, be denoted as ${\overset{⌢}{f}}_{\tau }$. Within a strictly deterministic setting, a smooth functional *f*(**x**) tends to become stationary, in the sense of a vanishing first variation, as **x** approaches an extremal value x*. In the stochastic setting, a counterpart of this scenario could be that any future conditioning of the process **x**_*τ*_ on $N_{\xi }$, which is the filtration generated by ${\overset{⌢}{f}}_{s}$, *s*<*ξ*, until a past iteration (i.e. *ξ*<*τ*), identically yields the random variable **x**_*ξ*_ itself, i.e. $E({\text{x}}_{\tau }|N_{\xi })={\text{x}}_{\xi }$, where *E* represents the expectation operator. Via the Markov structure of **x**_*τ*_ one may equivalently postulate that a necessary condition for the extremization of *f*_*τ*_:=*f*(**x**_*τ*_) is to require that *E*(**x**_*τ*_|${\overset{⌢}{f}}_{\xi }$)=**x**_*ξ*_. This characterization renders the process **x**_*τ*_ a martingale with respect to the extremal cost filtration $N_{\tau }$, i.e. once extremized, any future conditional mean of **x**_*τ*_ on the cost filtration remains iteration invariant. See [24] for an introductory treatise on the theory of martingales. An equivalent way of stating this condition is based on an error or ‘innovation’ process defined as ${\overset{⌢}{f}}_{\tau }$−*f*_*τ*_. An extremization would require updating **x**_*τ*_ to statistically match the extremal process ${\text{x}}_{\tau }^{*}$ so that the innovation ${\overset{⌢}{f}}_{\tau }-f({\text{x}}_{\tau }^{*})$ is driven to a zero mean martingale, e.g. a zero-mean Brownian motion or, more generally, a stochastic integral in Ito's sense (or, in a *τ*-discrete setting, a zero-mean random walk). This enables one to pose the determination of an extremum as a martingale problem, originally conceptualized by Stroock & Varadhan [20] to provide an improved setting for solutions to SDEs beyond Ito's theory. If the update scheme is effective, one anticipates ${\text{x}}_{\tau }\to {\text{x}}_{\tau }^{*}$ asymptotically, i.e. as τ→∞. In other words, $E(\lim_{\tau \to \infty }E({\text{x}}_{\tau }|N_{\tau }))=E({\text{x}}_{\tau }^{*})={\text{x}}^{*}$ and, as the norm of the noise intensity associated with **x**_*τ*_ approaches zero, ${\text{x}}_{\tau }^{*}\to {\text{x}}^{*}$. However, since strictly zero noise intensity is infeasible within the stochastic set-up, the solution in a deterministic setting may be thought of as a degenerate version (with a Dirac measure) of that obtained through the stochastic scheme.

Note that a ready extension of this approach is possible with multi-objective functions, which would merely require building up the innovation as a vector-valued process. Similarly, the approach may also be adapted for treating equality constraints, wherein zero-valued constraint functions, perturbed by zero-mean noise processes, may be used to construct the innovation processes. The easy adaptability of the current set-up for multiple objective functions or constraints may be viewed as an advantage over most existing evolutionary optimization schemes, where a single cost functional needs to be constructed based on all the constraints, a feature that may possibly engender instability for a class of large dimensional problems.

Driving **x**_*τ*_ to an $N_{\tau }$-martingale, or forcing the innovation process to a zero-mean martingale, may be readily achieved through a change of measures. In evolutionary algorithms, e.g. the GA, the accompanying change of measures requires the assignment of weights or fitness values to the current set of particles and a subsequent selection of those with higher fitness, e.g. by rejection sampling. For a better exploration of the sample space, the GA uses steps like ‘crossover’ and ‘mutation’. These steps are biologically inspired safeguards against an intrinsic limitation of the weight-based update, which tends to diminish all the weights but one to zero, thereby leaving the realized particles nearly identical after a few iterations. This problem is referred to as that of weight collapse or particle impoverishment and precipitates premature convergence to a wrong solution. As a way out of this degeneracy in the realized population set (i.e. the ensemble), the present scheme employs a purely additive update, derived following a Girsanov change of measures *P*→*Q* (see [19] for a detailed exposition on the Girsanov transformation), which ensures that the innovation process, originally described under measure *P*, becomes a zero-mean martingale under the new measure *Q*. A basic tool in effecting this change of measure is the Radon–Nikodym derivative *Λ*_*τ*_:=d*P*/d*Q* (assuming absolute continuity of *Q* w.r.t. *P* and vice versa), a scalar valued random variable also called the likelihood ratio (or the fitness or the weight) that multiplicatively updates the design process **x**_*τ*_ as **x**_*τ*_*Λ*_*τ*_. In order to bypass the degeneracy problem noted above, one may obtain an additive update by expanding **x**_*τ*_*Λ*_*τ*_ using Ito's formula [19]. Within an MC set-up, an immediate consequence of the additive update is that particles with lower weights are never eliminated, but rather corrected to have more fitness by being driven closer to an extremum. Drawing analogy with Taylor's expansion of a smooth function(al) that obtains its first-order term based on Newton's directional derivative, the present version of the additive correction may be interpreted as a non-Newton directional term that drives the innovation process to a zero-mean martingale and a precise form of this term is derived later in this section. In general, the innovation process may be a vector. One such case occurs when, by way of addressing possible numerical instability, a single cost functional is split into several so that each of the correspondent innovations is driven to a zero-mean martingale. This in turn ensures that the innovation corresponding to the original cost functional is also driven to a zero-mean martingale. For a simpler exposition of the basic ideas, the method below is presented using a single cost functional only. The formulation can be trivially extended for vector innovation processes of any finite dimension.

Within a *τ*-discrete framework with *τ*∈(*τ*_*i*−1_,*τ*_*i*_], **x**_*τ*_ is evolved following equation (2.1). An innovation constraint that may be satisfied for an accelerated (greedy) search in a neighbourhood of an extremum is given by

2.3 $${\overset{⌢}{f}}_{\tau }-f({\text{x}}_{\tau })=\Delta \eta_{\tau },$$

where ${\overset{⌢}{f}}_{\tau }\in R$ is the extremal cost process, *f* the objective functional and $\Delta \eta_{\tau }=\eta_{\tau }-\eta_{i-1}\in R$ a *P*-Brownian increment representing the diffusive fluctuations. Deriving the subsequent integro-differential equation for the search is best accomplished by recasting equation (2.3) as an SDE. Towards this, an $N_{\tau }$-measureable process ${\overset{⌣}{f}}_{\tau }$:=${\overset{⌣}{f}}_{\left(\tau \right)}$ may be constructed to arrive at the following incremental form:

2.4 $$\Delta {\overset{⌣}{f}}_{\tau }:={\overset{⌢}{f}}_{\tau }\Delta \tau =f({\text{x}}_{\tau })\Delta \tau +\Delta \eta_{\tau }\Delta \tau ;\Delta \tau =\tau -\tau_{i-1}.$$

Here Δ*τ*_*i*_:=*τ*_*i*_−*τ*_*i*−1_ is taken as a ‘small’ increment. Since the *τ*-axis is fictitious, Δ*τ* is replaced by Δ*τ*_*i*_=*τ*_*i*_−*τ*_*i*−1_, so that equation (2.4) is modified to

2.5 $$\Delta {\overset{⌣}{f}}_{\tau }=f({\text{x}}_{\tau })\Delta \tau +\Delta \eta_{\tau }\Delta \tau_{i},$$

which is essentially correspondent to the SDE:

2.6 $$d{\overset{⌣}{f}}_{\tau }=f({\text{x}}_{\tau })\;d\tau +\Delta \tau_{i}\;d\eta_{\tau }.$$

Note that the replacement of Δ*τ* by Δ*τ*_*i*_ merely modifies the intensity of the noise process Δ*η*_*τ*_ in equation (2.3). The form of SDE (2.6) has the desirable feature that the fictitious diffusion coefficient Δ*τ*_*i*_ is an order ‘smaller’ relative to the drift coefficient *f*(**x**_*τ*_). However, since *η*_*τ*_ is not standard Brownian, it is more convenient to rewrite equation (2.6) as

2.7 $$d{\overset{⌣}{f}}_{\tau }=f({\text{x}}_{\tau })\;d\tau +\rho_{\tau }\;dW_{\tau },$$

where *W*_*τ*_ is standard *P*-Brownian and *ρ*_*τ*_ a more general form of (scalar-valued) noise intensity that may be made a function of *τ*. For multi-objective optimization problems (where ${\overset{⌣}{f}}_{\tau }$ is a vector stochastic process), *ρ*_*τ*_ would be the intensity matrix and the expressions below are so written that they are valid for both scalar and vector cases. Equation (2.7) may be rewritten as

2.8 $$d{\tilde{f}}_{\tau }:=\rho_{\tau }^{-1}\;d{\overset{⌣}{f}}_{\tau }=h({\text{x}}_{\tau })\;d\tau +dW_{\tau },$$

where $h({\text{x}}_{\tau }):=\rho_{\tau }^{-1}f({\text{x}}_{\tau })$. While it is possible, and even desirable, to replace the Brownian noise term above by one whose quadratic variation is zero (e.g. a Poisson martingale), such a modification is not central to the basic idea and will be considered in future extensions of the work. SDE (2.7) is assumed to satisfy the standard conditions [19] so as to ensure the existence of a weak solution. The $N_{\tau }$-measurable locally optimal (extremal) solution may now be identified with the conditional mean $E({\text{x}}_{\tau }|N_{\tau })$. Considering a new measure *Q* under which **x**_*τ*_ from equation (2.1) satisfies equation (2.7), the conditional mean may be expressed via the generalized Bayes' formula as

2.9 $$\pi_{\tau }(\text{x}):=E({\text{x}}_{\tau }|N_{\tau })=\frac{E_{Q}({\text{x}}_{\tau }\Lambda_{\tau }|N_{\tau })}{E_{Q}(\Lambda_{\tau }|N_{\tau })},$$

where the expectation *E*_*Q*_(⋅) is taken with respect to the new measure *Q* and *Λ*_*τ*_ is the scalar fitness given by

2.10 $$\Lambda_{\tau }=\exp\left[\int_{\tau_{i-1}}^{\tau }h_{s}\;d{\tilde{f}}_{s}-\frac{1}{2}\int_{\tau_{i-1}}^{\tau }h_{s}^{T}h_{s}\;ds\right].$$

As shown in appendix A (a theorem and its corollary) using Ito's expansions of **x**_*τ*_*Λ*_*τ*_ and (*Λ*_*τ*_)^−1^, the incrementally additive updates to arrive at an extremum must follow from the differential equation:

2.11 $$d\pi_{\tau }(\text{x})=(\pi_{\tau }(\text{x}f)-\pi_{\tau }(\text{x})\pi_{\tau }(f)){\left(\rho_{\tau }\rho_{\tau }^{T}\right)}^{-1}(d{\overset{⌣}{f}}_{\tau }-\pi_{\tau }(f)\;d\tau )$$

and

2.12 $$\pi_{\tau }(\text{x})=\pi_{i-1}(\text{x})+\int_{\tau_{i-1}}^{\tau }(\pi_{s}(\text{x}f)-\pi_{s}(\text{x})\pi_{s}(f)){\left(\rho_{s}\rho_{s}^{T}\right)}^{-1}(d{\overset{⌣}{f}}_{s}-\pi_{s}(f)\;ds).$$

Note that the directionality of a search process provided by the second (integral) term on the right-hand side (r.h.s.) of the equation above may also be gauged from the fact the integrand in this term can be interpreted as a Malliavin-type derivative using Clarke–Ocone theorem [25]. But the appearance of the unknown term *π*_*τ*_(*f*) on the r.h.s. prevents equations (2.11) or (2.12) to be qualified as an SDE in *π*_*τ*_(**x**). Indeed, a necessarily nonlinear dependence of *f* on **x**_*τ*_ and the consequent non-Gaussianity of *π*_*τ*_(*f*) would prevent writing the latter in terms of *π*_*τ*_(**x**). This results in the so-called closure problem in solving for *π*_*τ*_(**x**).

While a direct solution of equations (2.11) or (2.12) yields an extremum (i.e. a local extremum only) in principle, exact/analytical solutions are infeasible owing to the circularity inherent in the closure problem. This has a parallel in nonlinear stochastic filtering, wherein the Kushner–Stratonovich equation [26] (an equivalent of equation (2.11)) also suffers from a similar circularity problem. Motivated by the MC filters used to solve nonlinear filtering problems [27,28], an MC scheme may be developed for a numerical treatment of equations (2.11) or (2.12). A two-stage strategy, viz. prediction and update, may apparently be considered, even though, as we will soon see, the prediction step could be entirely eliminated in the final scheme. The prediction step, as in most evolutionary optimization schemes, aims at an initial random exploration based on equation (2.1). Consider the *i*th iteration, i.e. *τ*∈(*τ*_*i*−1_,*τ*_*i*_] and let *N* denote the ensemble size so that one realizes *N* predicted particles or MC candidates, ${\left\{{\text{x}}_{\tau }^{\left(j\right)}\right\}}_{j=1}^{N}$ that must be updated via equation (2.12). For an MC-based numerical solution to equation (2.12), a sample-averaged form of the equation is first written as

2.13 $$\pi_{\tau }^{N}(\text{x})=\pi_{i-1}^{N}(\text{x})+\int_{\tau_{i-1}}^{\tau }(\pi_{s}^{N}(\text{x}f)-\pi_{s}^{N}(\text{x})\pi_{s}^{N}(\;f)){\left(\rho_{s}\rho_{s}^{T}\right)}^{-1}(d{\overset{⌣}{f}}_{s}-\pi_{s}^{N}(f)\;ds).$$

$\pi^{N}(.)=(1/N)\sum_{j=1}^{N}{\left(.\right)}^{\left(j\right)}$ is the ensemble-averaged approximation to the conditional mean *π*(.). A particle-wise representation of equation (2.13) is given by

2.14 $${\text{X}}_{\tau }={\text{X}}_{i-1}+\frac{1}{N}\int_{\tau_{i-1}}^{\tau }\left\{{\text{X}}_{s}{{\text{F}}_{s}}^{T}-{\hat{\text{X}}}_{s}{{\hat{\text{F}}}_{s}}^{T}\}{\left(\rho_{s}\rho_{s}^{T}\right)}^{-1}\{d{\overset{⌣}{\text{F}}}_{s}-{\text{F}}_{s}\;ds\right\},$$

where ${\text{X}}_{\tau }:=[{\text{x}}_{\tau }^{\left(1\right)},{\text{x}}_{\tau }^{\left(2\right)},\ldots {\text{x}}_{\tau }^{\left(N\right)}]$, ${\text{F}}_{\tau }:=[f_{\tau }^{\left(1\right)},f_{\tau }^{\left(2\right)},\ldots ,f_{\tau }^{\left(N\right)}]$, ${\hat{\text{X}}}_{\tau }:=\pi_{\tau }^{N}(\text{x})\text{r}\in R^{n\times N}$, ${\hat{\text{F}}}_{\tau }:=\pi_{\tau }^{N}(\;f)\text{r}\in R^{N}$ and $d{\overset{⌣}{\text{F}}}_{\tau }:=d{\overset{⌣}{f}}_{\tau }\text{r}\in R^{N}$. $\text{r}=\{1,\;1,\ldots ,1\}\in R^{N}$ is an *N*-dimensional row vector with entries 1. Note that the second term on the r.h.s. of equation (2.14) is the additive update/correction term. For solving equation (2.14), the MC approximation to equation (2.12), a *τ*-discrete numerical scheme is required. Such a scheme would typically involve the following two steps.

### 2.1 Prediction

The predicted particle set ${\tilde{\text{X}}}_{\tau }=[{\tilde{\text{x}}}_{\tau }^{\left(1\right)},\ldots ,{\tilde{\text{x}}}_{\tau }^{\left(N\right)}]$ at *τ* is generated using an Euler–Maruyama (EM) discretized map

2.15 $${\tilde{\text{X}}}_{\tau }={\text{X}}_{i-1}+\Delta \Psi_{\tau },$$

where $\Delta \Psi_{\tau }:=[\Delta \xi_{\tau }^{\left(1\right)},\ldots ,\Delta \xi_{\tau }^{\left(N\right)}]$, $\Delta \xi_{\tau }^{\left(1\right)}=\xi_{\tau }^{\left(1\right)}-\xi_{i-1}^{\left(1\right)}$, etc.

### 2.2 Additive update

The update to the predicted particles is through an EM approximation to the integral in equation (2.14). Higher order integration schemes could also be considered, especially for evaluating the correction integral. The discrete update equation is presently given by

2.16 $${\text{X}}_{\tau }={\tilde{\text{X}}}_{\tau }+\frac{1}{N}\left\{{\tilde{\text{X}}}_{\tau }{\tilde{\text{F}}}_{\tau }^{T}-{\tilde{\hat{\text{X}}}}_{\tau }{\tilde{\hat{\text{F}}}}_{\tau }^{T}\right\}{\left(\rho_{\tau }\rho_{\tau }^{T}\right)}^{-1}\left\{\Delta {\overset{⌣}{\text{F}}}_{\tau }-{\tilde{\text{F}}}_{\tau }\Delta \tau \right\},$$

where Δ${\overset{⌣}{F}}_{\tau }$=Δ${\overset{⌣}{f}}_{\tau }$**r**, ${\tilde{\text{F}}}_{\tau }:=[{\tilde{f}}_{\tau }^{\left(1\right)},\ldots ,{\tilde{f}}_{\tau }^{\left(N\right)}]$ and the predicted solution, ${\tilde{\text{x}}}_{\tau }$, is used to compute ${\tilde{f}}_{\tau }:=f({\tilde{\text{x}}}_{\tau })$. Moreover, ${\tilde{\hat{\text{F}}}}_{\tau }:=\pi_{\tau }^{N}(\tilde{f})\text{r}$. Equation (2.16) may be recast as

2.17 $${\text{X}}_{\tau }={\tilde{\text{X}}}_{\tau }+\frac{1}{N}\left\{({\tilde{\text{X}}}_{\tau }-{\tilde{\hat{\text{X}}}}_{\tau }){{\tilde{\text{F}}}_{\tau }}^{T}+{\tilde{\hat{\text{X}\;}\;\;}\;}_{\tau }{\left({\tilde{\text{F}}}_{\tau }-{\tilde{\hat{\text{F}}}}_{\tau }\right)}^{T}\right\}{\left(\rho_{\tau }\rho_{\tau }^{T}\right)}^{-1}\left\{\Delta {\overset{⌣}{\text{F}}}_{\tau }-{\tilde{\text{F}}}_{\tau }\Delta \tau \right\}.$$

Recall from equation (2.4) that Δ${\overset{⌣}{f}}_{\tau }$:=${\overset{⌢}{f}}_{\tau }$Δ*τ* so that equation (2.17) may be rearranged as

2.18 $${\text{X}}_{\tau }={\tilde{\text{X}}}_{\tau }+\frac{1}{N}\left\{({\tilde{\text{X}}}_{\tau }-{\tilde{\hat{\text{X}}}}_{\tau })({\tilde{\text{F}}}_{\tau }^{T}\Delta \tau )+({\tilde{\hat{\text{X}}}}_{\tau }\Delta \tau ){\left({\tilde{\text{F}}}_{\tau }-{\tilde{\hat{\text{F}}}}_{\tau }\right)}^{T}\right\}{\left(\rho_{\tau }\rho_{\tau }^{T}\right)}^{-1}\left\{{\overset{⌢}{\text{F}}}_{\tau }-{\tilde{\text{F}}}_{\tau }\right\},$$

where ${\overset{⌢}{\text{F}}}_{\tau }={\overset{⌢}{f}}_{\tau }\text{r}\in R^{N}$. When the particles are somewhat away from a local extremum (e.g. during the initial stages of evolution), the (norm of the) correction term is large. Hence the particles traverse more in the search space. In such cases, the innovation process would not behave as a zero-mean martingale as it would have a significant drift component. Since evolutions in this regime may have sharper *τ*-gradients, it is appropriate to modify the coefficient matrix in equation (2.18) so as to incorporate information on these gradients through previous estimates. Thus, ${\tilde{\text{F}}}_{\tau }^{T}\Delta \tau$ and ${\tilde{\hat{\text{X}}}}_{\tau }\Delta \tau$ are replaced, respectively, by the following approximations:

2.19 $${\tilde{\text{F}}}_{\tau }^{T}\Delta \tau \approx ({\tilde{\text{F}}}_{\tau }^{T}\tau -{\tilde{\hat{\text{F}}}}_{i-1}^{T}\tau_{i-1}-\Delta {\tilde{\hat{\text{F}}}}_{\tau }^{T}\tau )$$

and

2.20 $${\tilde{\hat{\text{X}}}}_{\tau }\Delta \tau \approx ({\tilde{\hat{\text{X}}}}_{\tau }\tau -{\tilde{\hat{\text{X}}}}_{i-1}\tau_{i-1}).$$

Note that we have used Ito's formula while approximating the r.h.s. in equation (2.19). Using equations (2.19) and (2.20), equation (2.18) may be modified as

2.21 $${\text{X}}_{\tau }={\tilde{\text{X}}}_{\tau }+\frac{1}{N}\left\{\begin{array}{c} c({\tilde{\text{X}}}_{\tau }-{\tilde{\hat{\text{X}}}}_{\tau })({\tilde{\text{F}}}_{\tau }^{T}\tau -{\tilde{\hat{\text{F}}}}_{i-1}^{T}\tau_{i-1}-\Delta {\tilde{\hat{\text{F}}}}_{\tau }^{T}\tau )\\ \;+({\tilde{\hat{\text{X}}}}_{\tau }\tau -{\tilde{\hat{\text{X}}}}_{i-1}\tau_{i-1}){\left({\tilde{\text{F}}}_{\tau }-{\tilde{\hat{\text{F}\;}\;\;}\;}_{\tau }\right)}^{T} \end{array}\right\}{\left(\rho_{\tau }\rho_{\tau }^{T}\right)}^{-1}\{{\overset{⌢}{\text{F}}}_{\tau }-{\tilde{\text{F}\;}\;}_{\tau }\}.$$

Once the converged estimate is obtained, the innovation noise covariance $\rho_{\tau }\rho_{\tau }^{T}$ should satisfy the identity:

2.22 $$\begin{array}{rl} \rho_{\tau }\rho_{\tau }^{T} & \approx \pi_{\tau }^{N}(({\overset{⌢}{f}}_{\tau }-{\tilde{f}}_{\tau }){\left({\overset{⌢}{f}}_{\tau }-{\tilde{f}}_{\tau }\right)}^{T})\\ & =\frac{1}{N-1}(\left({\overset{⌢}{\text{F}}}_{\tau }-{\tilde{\text{F}}}_{\tau })-({\overset{⌢}{\text{F}}}_{\tau }-{\tilde{\hat{\text{F}}}}_{\tau }\right)){\left(\left({\overset{⌢}{\text{F}}}_{\tau }-{\tilde{\text{F}}}_{\tau })-({\overset{⌢}{\text{F}}}_{\tau }-{\tilde{\hat{\text{F}}}}_{\tau }\right)\right)}^{T}. \end{array}$$

Prior to convergence to an extremum, the (norm of the) r.h.s. of the equation above would typically be relatively large. Thus, one could impart higher diffusion to the search in the initial stages by replacing $\rho_{\tau }\rho_{\tau }^{T}$ in equation (2.21) by

2.23 $$\alpha \frac{1}{N-1}(({\overset{⌢}{\text{F}}}_{\tau }-{\tilde{\text{F}}}_{\tau })-({\overset{⌢}{\text{F}}}_{\tau }-{\tilde{\hat{\text{F}}}}_{\tau })){\left(({\overset{⌢}{\text{F}}}_{\tau }-{\tilde{\text{F}}}_{\tau })-({\overset{⌢}{\text{F}}}_{\tau }-{\tilde{\hat{\text{F}}}}_{\tau })\right)}^{T}+(1-\alpha )\rho_{\tau }\rho_{\tau }^{T}.$$

Here *α*∈(0,1]. It is typically taken as 0.8 in the numerical illustrations based on *pseudo-codes 1, 2* and *3*. Equation (2.21) thus takes the final form:

2.24 $$\begin{array}{rl} {\text{X}}_{\tau } & ={\tilde{\text{X}}}_{\tau }+\frac{1}{N}\left\{\begin{array}{l} c\left({\tilde{\text{X}}}_{\tau }-{\tilde{\hat{\text{X}}}}_{\tau })({\tilde{\text{F}}}_{\tau }^{T}\tau -{\tilde{\hat{\text{F}}}}_{i-1}^{T}\tau_{i-1}-\Delta {\tilde{\hat{\text{F}}}}_{\tau }^{T}\tau \right)\\ \;+({\tilde{\hat{\text{X}}}}_{\tau }\tau -{\tilde{\hat{\text{X}}}}_{i-1}\tau_{i-1}){\left({\tilde{\text{F}}}_{\tau }-{\tilde{\hat{\text{F}}}}_{\tau }\right)}^{T} \end{array}\right\}\\ & \;\times {\left\{\begin{array}{c} c\alpha \frac{1}{N-1}(\left({\overset{⌢}{\text{F}}}_{\tau }-{\tilde{\text{F}}}_{\tau })-({\overset{⌢}{\text{F}}}_{\tau }-{\tilde{\hat{\text{F}}}}_{\tau }\right)).\\ {\left(({\overset{⌢}{F}}_{\tau }-{\tilde{F}}_{\tau })-({\overset{⌢}{F}}_{\tau }-{\tilde{\hat{F}}}_{\tau })\right)}^{\text{T}}+(1-\alpha )\rho_{\tau }\rho_{\tau }^{T} \end{array}\right\}}^{-1}\{{\overset{⌢}{\text{F}}}_{\tau }-{\tilde{\text{F}}}_{\tau }\}. \end{array}$$

A more concise form of the update equation is

2.25 $${\text{X}}_{\tau }={\tilde{\text{X}}}_{\tau }+{\tilde{\text{G}}}_{\tau }\{{\overset{⌢}{\text{F}}}_{\tau }-{\tilde{\text{F}}}_{\tau }\},$$

where the gain-like update coefficient matrix is given by

$$\begin{array}{rl} {\tilde{\text{G}}}_{\tau } & :=\frac{1}{N}\left\{\begin{array}{c} c({\tilde{\text{X}}}_{\tau }-{\tilde{\hat{\text{X}}}}_{\tau })({\tilde{\text{F}}}_{\tau }^{T}\tau -{\tilde{\hat{\text{F}}}}_{i-1}^{T}\tau_{i-1}-\Delta {\tilde{\hat{\text{F}}}}_{\tau }^{T}\tau )\\ \;+({\tilde{\hat{\text{X}}}}_{\tau }\tau -{\tilde{\hat{\text{X}}}}_{i-1}\tau_{i-1}){\left({\tilde{\text{F}}}_{\tau }-{\tilde{\hat{\text{F}}}}_{\tau }\right)}^{T} \end{array}\right\}\\ & \;\times {\left\{c\alpha \frac{1}{N-1}(({\overset{⌢}{\text{F}}}_{\tau }-{\tilde{\text{F}}}_{\tau })-({\overset{⌢}{\text{F}}}_{\tau }-{\tilde{\hat{\text{F}}}}_{\tau })){\left(({\overset{⌢}{\text{F}}}_{\tau }-{\tilde{\text{F}}}_{\tau })-({\overset{⌢}{\text{F}}}_{\tau }-{\tilde{\hat{\text{F}}}}_{\tau })\right)}^{T}+(1-\alpha )\rho_{\tau }\rho_{\tau }^{T}\right\}}^{-1}. \end{array}$$

In the update strategy of equation (2.25), one may still improve on the search space exploration by multiplying the gain-weighted innovation term by a scalar factor *β*_*τ*_<1. This is equivalent to increasing the noise covariance and hence allowing the particles to be more diffusive or ‘explorative’. Thus, the update equation becomes

2.26 $${\text{X}}_{\tau }={\tilde{\text{X}}}_{\tau }+\beta_{\tau }{\tilde{\text{G}}}_{\tau }\{{\overset{⌢}{\text{F}}}_{\tau }-{\tilde{\text{F}}}_{\tau }\}.$$

## 3. Coalescence, scrambling and relaxation: schemes for global search

The possible trapping of particles in local extrema is a major challenge to any global optimization scheme. For *β*_*τ*_≥1, the scheme described in equation (2.26) yields a greedy search that may often end up in a local extremum. Although it seems possible to avoid the local traps through the innovation noise, whose intensity could be tuned by the scalar factor *β*_*τ*_, such an approach may not be quite effective. As a local extremum is approached, the ‘strength’ (or norm) of the update term would be small and consequently its sensitivity to variations in *β*_*τ*_ would also be poor. This makes the choice of *β*_*τ*_ difficult (e.g. necessitating *β*_*τ*_ to be too small for the search scheme to be efficient) and less effective for the global search. Moreover, smaller *β*_*τ*_ also implies larger diffusion and hence poorer convergence. A more effective way out is a random perturbation applied to the particles so as to force out the ones trapped in the local wells. A general perturbation scheme, combining three basic approaches referred to as ‘coalescence’, ‘scrambling’ and ‘relaxation’, is now described. While the coalescence component is implemented through yet another martingale problem, the scrambling part requires a perturbation kernel. The relaxation component, on the other hand, requires accepting improvements with some probability. The importance of the random perturbation steps, in the present context, may also be gauged from the following fact. For a class of problems considered later, the faster convergence to a local extremum, engendered by the greedy scheme, equation (2.26), may force wrong convergence despite the application of random perturbations in conjunction with the greedy local search. Hence it would be worth exploring alternative search schemes, which completely eliminate scheme (2.26) and rely on the random perturbation steps alone.

Since the aim is to pose ‘coalescence’ as a martingale problem, the associated update should share the same generic structure as equation (2.26), which is recast below for the *j*th candidate

3.1 $${\text{x}}_{i}^{\left(j\right)}={\tilde{\text{x}}}_{i}^{\left(j\right)}+{\tilde{\text{C}}}_{i}^{\left(j\right)},$$

where ${\tilde{\text{C}}}_{i}^{\left(j\right)}:=\beta_{i}{\tilde{\text{G}}}_{i}\{{\overset{⌢}{f}}_{i}-{\tilde{f}}_{i}^{\left(j\right)}\}$. The basic idea here is to provide to the evolving solution layers of random perturbation, whose inverse intensity may be formally indexed by a positive integer *l* such that the perturbation vanishes as l→∞. Within the *τ*-discrete setting, we start with the prediction ${\tilde{\text{x}}}_{i}$ and denote by ${\;}^{l}\Delta {\hat{\text{x}}}_{i}={\;}^{l}{\text{x}}_{i}-{\;}^{l}{\tilde{\text{x}}}_{i}$ the randomly perturbed, *l*-indexed increment so that ${\;}^{\infty }\Delta {\hat{\text{x}}}_{i}\to \Delta {\text{x}}_{i}$ as the random perturbations vanish asymptotically. During the *i*th iteration, the perturbed increment is arrived at using two transitional increments, ^*l*^**u**_*i*_ and ^*l*^**v**_*i*_ determined by two perturbation operators, say **T1** and **T2**, respectively. While the operator **T1** corresponds to the ‘local search’ and/or ‘coalescence’ operations, **T2** encapsulates the ‘scrambling’ and ‘relaxation’ operations. The transitions may be indicated as ${\;}^{l}\Delta {\text{x}}_{i}\to \lim_{\text{T1}}{\;}^{l}{\text{u}}_{i}\to \lim_{\text{T2}}{\;}^{l}{\text{v}}_{i}\to \lim_{\text{T3}}{\;}^{l}\Delta {\hat{\text{x}}}_{i}$, where **T3** is a selection operator, commonly used with most evolutionary optimization schemes in some form or the other. Here ${\;}^{l}\Delta {\hat{\text{x}}}_{i}$ is the final increment at *τ*_*i*_, i.e. ${\;}^{l}{\text{x}}_{i}:={\;}^{l}{\tilde{\text{x}}}_{i}+{\;}^{l}\Delta {\hat{\text{x}}}_{i}$, which is input to the next iteration. ${\;}^{l}\Delta {\text{x}}_{i}={\;}^{l}{\tilde{\text{x}}}_{i}-{\;}^{l}{\text{x}}_{i-1}$ denotes the predicted increment. Ideally, one may start the iterations with small *l* (i.e. high perturbation intensity), which is then gradually increased with progressing iterations. However, in the current implementations of COMBEO, the perturbation intensity is kept uniform all through the iterations. This is possible as, upon convergence to the global optimum, the applied perturbations merely yield zero-mean random fluctuations about the optimum, which are averaged out when the sample expectation operation is performed in the MC simulation. In the view of this and for notational ease, the left superscript *l* is removed from the variables in the discussion to follow and the perturbed nature of the variables should be clear from the context. The operators are now defined below.

### 3.1 Operator **T1**: local search and coalescence

The operation for local search has been described in §2 and through equation (3.1). The operation of ‘coalescence’ is now outlined. This perturbation is motivated by the observation that the probability density function (PDF, if it exists) associated with the converged measure *π*_*τ*_(.) should be unimodal, with its only peak located at the global extremum. Thus, when convergence to the global extremum occurs, all the particles should coalesce at the optimum point, except for a zero-mean noisy scatter around the latter. Ideally, for the sake of optimization accuracy, the noisy scatter should also have a low intensity so as to keep sample fluctuations under control. Once the global optimization scheme converges, the noisy scatter should then behave as a zero-mean martingale as a function of *τ* and with a unimodal transitional PDF. A zero-mean Brownian motion, which has a Gaussian PDF, is one such martingale. Clearly, such a property does not hold away from the global optimum, where the PDF would be multi-modal with a peak located at every local extremum detected by the algorithm.

Now, the aim is to obtain a scheme (or rather a family of schemes) to force the coalescence of particles. Consider the update of the *j*th particle ${\text{x}}_{\tau }^{\left(j\right)}$ such that coalescence of particles can be enforced. The random scatter around ${\text{x}}_{\tau }^{\left(j\right)}$ could be quantified by $\delta_{\tau }(j)={\text{x}}_{\tau }^{\left(\sigma_{1}(j)\right)}-{\text{x}}_{\tau }^{\left(j\right)}$, where ***σ***_1_(*j*) denotes a random permutation on the indexing set {1,*N*}∖{*j*} based on a uniform measure. The goal is then to drive $\delta_{\tau }(j)={\text{x}}_{\tau }^{\left(\sigma_{1}(j)\right)}-{\text{x}}_{\tau }^{\left(j\right)}$ to a zero-mean vector Brownian increment $\Delta \eta_{\tau }^{c}$ with intensity matrix $\rho_{\tau }^{c}$ (typically assumed to be diagonal with the entries chosen uniformly for all *j*). In this case, ‘coalescence’ then implies that, in the limit of the noise intensity in *δ*_*τ*_(*j*) approaching zero, all the particles would tend to coalesce into a single location at the global extremum. Thus, similar to the innovation ${\overset{⌢}{f}}_{\tau }$−*f*(**x**_*τ*_) on the left-hand side of equation (2.3), one treats ${\text{x}}_{\tau }^{\left(\sigma_{1}(j)\right)}-{\text{x}}_{\tau }^{\left(j\right)}$ as yet another innovation process so that, upon convergence, the identity ${\text{x}}_{\tau }^{\left(\sigma_{1}(j)\right)}-{\text{x}}_{\tau }^{\left(j\right)}=\Delta \eta_{\tau }^{c}$ holds almost surely. Here, $\Delta \eta_{\tau }^{c}$ is responsible for an additional layer of randomness that gives every particle ${\text{x}}_{\tau }^{\left(j\right)}$ the structure of a stochastic process. Accordingly, the extremal cost filtration $N_{\tau }$ must be suitably expanded/modified to include the sub-filtration generated by $\Delta \eta_{s}^{c}$ for *s*≤*τ*. If the coalescence innovation, in the form as indicated above, is included within our search process, equation (3.1) must be modified as

3.2 $${\text{x}}_{i}^{\left(j\right)}={\tilde{\text{x}}}_{i}^{\left(j\right)}+{\tilde{\text{D}}}_{i}^{\left(j\right)}\;\text{or}\;{\text{u}}_{i}^{\left(j\right)}={\tilde{\text{D}}}_{i}^{\left(j\right)},$$

where

3.3 $${\tilde{\text{I}}}_{i}^{\left(j\right)}:=\begin{array}{c} {\tilde{\text{D}}}_{i}^{\left(j\right)}:=\beta_{i}{\tilde{\text{G}}}_{i}{\tilde{\text{I}}}_{i}^{\left(j\right)}\\ \left\{\begin{array}{c} c{\overset{⌢}{f}}_{i}-{\tilde{f}}_{i}^{\left(j\right)}\\ {\tilde{\text{x}}}_{i}^{\left(\sigma_{1}(j)\right)}-{\tilde{\text{x}}}_{i}^{\left(j\right)} \end{array}\right\}. \end{array}$$

Recall that over-tildes indicate either the predicted particles or functions evaluated using the predicted particles, as appropriate. With a convenient abuse, the same notation for the gain-like update coefficient matrix ${\tilde{\text{G}}}_{i}$ is retained in equation (3.3) (used earlier in the local update equation (2.25)). ${\tilde{\text{G}}}_{i}$ may now be computed as

3.4 $$\begin{array}{rl} {\tilde{\text{G}}}_{i} & :=\frac{1}{N}\left\{\begin{array}{c} c({\tilde{\text{X}}}_{i}-{\tilde{\hat{\text{X}}}}_{i})({\tilde{\text{F}}}_{i}^{T}\tau_{i}-{\tilde{\hat{\text{F}}}}_{i-1}^{T}\tau_{i-1}-\Delta {\tilde{\hat{\text{F}}}}_{i}^{T}\tau_{i})\\ \;+({\tilde{\hat{\text{X}}}}_{i}\tau_{i}-{\tilde{\hat{\text{X}}}}_{i-1}\tau_{i-1}){\left({\tilde{\text{F}}}_{i}-{\tilde{\hat{\text{F}}}}_{i}\right)}^{T} \end{array}\right\}{\left\{\begin{array}{c} c\alpha \frac{1}{N-1}(({\overset{⌢}{\text{F}}}_{i}-{\tilde{\text{F}}}_{i})-({\overset{⌢}{\text{F}}}_{i}-{\tilde{\hat{\text{F}}}}_{i}))\\ \;\cdot {\left(({\overset{⌢}{\text{F}}}_{i}-{\tilde{\text{F}}}_{i})-({\overset{⌢}{\text{F}}}_{i}-{\tilde{\hat{\text{F}}}}_{i})\right)}^{T}+(1-\alpha )\gamma_{i}\gamma_{i}^{T} \end{array}\right\}}^{-1} \end{array}$$

and

3.5 $$\gamma_{i}\gamma_{i}^{T}:=\left[\begin{array}{cc} \rho_{i}\rho_{i}^{T} & \text{0}\\ \text{0} & \rho_{i}^{c}{\left(\rho_{i}^{c}\right)}^{T} \end{array}\right].$$

Here, the *j*th column of ${\tilde{\text{F}}}_{i}$ is given as $\left\{\begin{array}{c} {\overset{⌢}{f}}_{i}-{\tilde{f}}_{i}^{\left(j\right)}\\ {\tilde{\text{x}}}_{i}^{\sigma_{1}(j)}-{\tilde{\text{x}}}_{i}^{\left(j\right)} \end{array}\right\}$. Let *a* be a positive real number and define the nearest integer smaller than *a* by ⌊*a*⌋. Then the integer valued perturbation parameter for the local extremization cum coalescence step may be identified as $l=\lfloor ∥{\left[\gamma_{i}\gamma_{i}^{T}\right]}^{-1}∥\rfloor$.

A real strength of the coalescence step is in the non-unique choice of the innovation vector—a feature that enables one to design powerful global search schemes. Indeed, one may also borrow some of the basic concepts from different existing global optimization schemes and adapt them within the present coalescence framework. For example, the personal and global information used in the PSO may be incorporated here by constructing the innovation as given below:

3.6 $${\tilde{\text{I}}}_{i}^{\left(j\right)}=\left\{\begin{array}{c} {\;}^{p}{\tilde{\text{x}}}_{i}^{\left(j\right)}-{\tilde{\text{x}}}_{i}^{\left(j\right)}\\ {\;}^{g}{\tilde{\text{x}}}_{i}-{\tilde{\text{x}}}_{i}^{\left(j\right)} \end{array}\right\}.$$

Here ${\;}^{p}{\tilde{\text{x}}}_{i}^{\left(j\right)}$ is the personal best location corresponding to the *j*th particle along its evolution history until *τ*_*i*_. ${\;}^{g}{\tilde{\text{x}}}_{i}$ is the available best location among all the particles in the population (ensemble) until *τ*_*i*_, i.e. during the same evolution history. In contrast to the PSO, the gain-type coefficient matrix here is founded on a sound probabilistic basis and hence its matrix structure enables iteration-dependent differential weighting of the scalar components of the innovation vector, thereby yielding faster convergence without losing the exploratory efficacy of the global search. Indeed, it is well recognized that the use of three parameters (e.g. the cognitive and social learning factors and the inertia weight) in the original PSO may lead to solutions that sensitively depend upon the choice of these parameters. Even though it is possible to incorporate within the current set-up, the basic ideas behind some of the augmented PSO schemes, which attempt at removing some of the shortfalls of the original PSO [29], such a detailed exercise is kept outside the ambit of the current work. While we do not provide a proof here, the convergence and uniqueness of the iterative increment through this step, for a non-decreasing sequence of *l* converging to a limit point l* which may be large, could be shown based on the seminal work of Stroock & Varadhan [20] on martingale problems.

### 3.2 Operator **T2**: scrambling and relaxation

Similar to **T1**, the operator **T2** also corresponds to random perturbations of the particles in the population. Identifying the gain-like coefficient matrix in the update equation (3.1) as a derivative-free stochastic counterpart to the Frechet derivative, the term ${\tilde{\text{C}}}_{i}^{\left(j\right)}$ may be considered the equivalent of a directional derivative term responsible for updating the *j*th particle. Consequently, around any extremum, the *L*^2^(*P*) norm $∥{\tilde{\text{C}}}_{i}^{\left(j\right)}∥$ is likely to be small. This has the effect of rendering further updates of the *j*th particle small, leading to a possible stalling of the optimization scheme. In order to move out of these local traps, a possible way is to swap the gain-weighted directional information for the *j*th particle with that of another randomly selected one. This form of random perturbation at *τ*_*i*_ may be implemented by replacing the update equation, e.g. equation (3.2), by any one of the following two perturbed equations:

3.7 $${\text{x}}_{i}^{\left(j\right)}={\tilde{\text{x}}}_{i}^{\left(j\right)}+{\tilde{\text{D}}}_{i}^{\sigma_{2}(j)}$$

or

3.8 $${\text{x}}_{i}^{\left(j\right)}={\tilde{\text{x}}}_{i}^{\sigma_{2}(j)}+{\tilde{\text{D}}}_{i}^{\left(j\right)},$$

where ${\tilde{\text{D}}}_{i}^{\left(j\right)}$ is update vector originally computed for the *j*th particle via equation (3.2) and ***σ***_2_ a random permutation on the integer set {1,*N*}. Out of the two perturbed equations above to implement scrambling, equation (3.8) is presently adopted. Formally, this perturbation may be described by a probability kernel *p*_*l*_ on [1,*N*]×[1,*N*] such that: $\sum_{i\in [1,N]}p_{l}(i,j)=1\forall \;j\in [1,N]$. Clearly, as l→∞ (or as τ→∞, i.e. as convergence to the global optimum takes place), the matrix *p*_*l*_(*i*,*j*) should ideally approach the identity matrix, i.e. *p*_*l*_(*i*,*j*)→*δ*_*ij*_, where *δ*_*ij*_ is the Kronecker delta.

By borrowing a basic idea from the DE and probably at the cost of a somewhat slower convergence for a class of problems, a more effective modification of the above scrambling strategy may be contemplated with a view to ensuring that the particles explore even more in the search space. Noting that every particle is an *n*-dimensional vector, one may execute the scrambling operation separately (*n* times) for individual scalar components of the particles instead of swapping the update terms for the particles as a whole. This may further be followed up by a so-called relaxation strategy, wherein the resulting modification is accepted with some probability *γ*<1. This improvement, enabling element crossovers across different particles, allows the particles to assume larger variations and prompt them to explore more. The resulting update equation is then given by

3.9 $$x_{i}^{m,(j)}={\tilde{x}}_{i}^{m,\sigma_{2}(j)}+{\tilde{D}}_{i}^{m,(j)},\;m\in [1,n],$$

where, for instance, $x_{i}^{m,(j)}$ denotes the updated *m*th scalar component of the *j*th particle at *τ*_*i*_. Since, upon convergence (and possibly owing to the coalescence step, if applied), all particles crowd around the peak of a unimodal PDF with progressing iterations, directional scrambling with relaxation across such converged particles should not, in any way, affect the numerical accuracy of the estimated global extremum. Hence, in practical implementations of our scheme, *p*_*l*_(*i*,*j*) need not strictly approach the identity matrix for large *l*.

### 3.3 Operator **T3**: selection

Use of diffusion-based random perturbations during exploration might sometimes result in ‘bad’ candidates. This may necessitate a selection step wherein candidates for the next iteration (say, the *i*th iteration) are chosen based on some selection criteria quantified by a selection or fitness function *g*(***υ***|**x**_*i*−1_), ***υ***∈*Ω*. A general construction of the function, which is a Markov transition kernel on *Ω* and is conditioned on the ensemble of particles at the last iteration, should satisfy the following properties:

(i) $g(\upsilon ={\text{x}}_{i}^{\left(j\right)}|{\text{x}}_{i-1}={\text{x}}_{i-1}^{\left(k\right)})=0$ if *j*≠*k*; ∀ *j*,*k*∈[1,*N*],
(ii) $g(\upsilon ={\text{x}}_{i}^{\left(j\right)}|{\text{x}}_{i-1}={\text{x}}_{i-1}^{\left(j\right)},f({\text{x}}_{i}^{\left(j\right)})\geq f({\text{x}}_{i-1}^{\left(j\right)}))=\varsigma \forall \;j\in [1,N],$ where *ς*∈(0,1], and
(iii) $g(\upsilon ={\text{x}}_{i-1}^{\left(j\right)}|{\text{x}}_{i-1}={\text{x}}_{i-1}^{\left(j\right)},f({\text{x}}_{i}^{\left(j\right)})<f({\text{x}}_{i-1}^{\left(j\right)}))=\varsigma$.

The updated *j*th particle appearing in the above clauses is computed using equation (3.7) (or equation (3.8)), which may incorporate different combinations of the three operations, e.g. local extremization, coalescence and scrambling-cum-relaxation. For the operator **T3**, the integer-valued inverse-perturbation parameter *l* may be identified with *l*=⌊1/(1−*ς*)⌋. In the current numerical implementations of COMBEO as described in §4, *ς*=1 is consistently adopted. This corresponds to *l* being infinity across all iterations and implies that the selection procedure is deterministic.

This work has primarily been aimed at the proposal for a class of new evolutionary optimization schemes and a verification of its performance purely through the numerical route. Accordingly, a detailed convergence analysis of the asymptotic dynamics, based on a combination of the martingale theory of Stroock–Varadhan and the random perturbation theory of Freidlin–Wentzell, will be considered in a separate study.

## 4. Algorithm development

The local search and random perturbation approaches, as described in §§2 and 3, merely provide a set of general tools whose different combinations could lead to different evolutionary schemes for optimization. For instance, incorporation of the innovation term in equation (2.3) yields a greedy algorithm that, despite possibly faster convergence, is likely to be a poor performer in the global search. On the other hand, to facilitate a more exhaustive global search at the cost of a substantively slower convergence, only a PSO-type innovation as in equation (3.6) could be adopted. For better clarity and a more objective assessment of the strengths and demerits of different search tools, three pseudo-codes are presented in this section. A more ambitious alternative would have been to combine all the presented ideas in a single pseudo-code that could automatically offer a right mix of convergence speed with exploratory efficiency depending on the nature of the problem at hand. This exercise however needs a non-trivial extension of the current work and hence constitutes an interesting future problem at this stage. *pseudo-code 1* uses the innovation term of equation (2.3) for a greedy local search while employing scrambling of particles as a whole (not element wise) for the global search. Importantly, it may be noted that while the prediction step of equation (2.1) appears to be helpful in exploration, this step is not practically useful with our schemes, especially as it does not exploit any directional information and as more efficient tools for explorations have already been laid out. Hence, given a random initial scatter to the particles provided at the beginning of the iterations, the prediction step has been completely eliminated from all subsequent iterations. This would typically mean that the number of evaluations of the objective functional would be reduced by half. Note that, as there is no prediction step, the over-tildes in the notations of the variables are henceforth removed.

**COMBEO** *pseudo-code 1*

1. Discretize the *τ*-axis, say $\left[\tau_{\min},\tau_{\max}\right]$, using a partition $\left\{\tau_{0}=\tau_{\min},\tau_{1},\ldots ,\tau_{M}=\tau_{\max}\right\}$ such that *τ*_0_<⋯<*τ*_*M*_ and *τ*_*i*_−*τ*_*i*−1_=Δ*τ*_*i*_ (=Δ*τ*:=1/*M* if a uniform step-size is chosen for *i*=0,…,*M*−1). Assign *τ*_0_=1 and adopt Δ*τ* small (approx. 10^−7^). Choose an ensemble size *N*.
2. From the domain of definition randomly generate, following a uniform distribution, the ensemble of initial particles (the initial population) ${\left\{{\text{x}}_{0}^{\left(j\right)}\right\}}_{j=1}^{N}$ for the solution vector. For each discrete *τ*_*i*_, *i*=1,…,*M*−1, execute the following steps.
3. *Additive update*
  Choose *α*∈(0,1); a typical prescription could be *α*≈0.8, even though the method generally performs well for other values in the interval indicated. Update each particle as
  $${\text{x}}_{i}^{\left(j\right)}={\text{x}}_{i-1}^{\sigma_{2}(j)}+{\text{D}}_{i}^{\left(j\right)},\;j=1,\ldots ,N,$$
  where ${\text{D}}_{i}^{\left(j\right)}$ is the *i*th correction vector. ***σ***_2_(*j*) is the *j*th random permutation based on a uniformly distributed probability measure over the integer set{1,…,*N*}. For convenience, the expression for ${\tilde{\text{D}}}_{i}^{\left(j\right)}$ is reproduced below:
  $$\begin{array}{rl} {\text{D}}_{i}^{\left(j\right)} & :=\beta_{i}{\text{G}}_{i}{\text{I}}_{i}^{\left(j\right)},\;j=1,\ldots ,N\\ {\text{I}}_{i}^{\left(j\right)} & :=\left\{\begin{array}{c} {\overset{⌢}{f}}_{i-1}-f_{i-1}^{\left(j\right)}\\ {\text{x}}_{i-1}^{\sigma_{1}(j)}-{\text{x}}_{i-1}^{\left(j\right)} \end{array}\right\}, \end{array}$$
  $\beta_{i}={\hat{\beta }}_{i}\zeta_{i}$ (${\hat{\beta }}_{i}$ is a scalar constant and *ζ*_*i*_ a uniform random number between 0 and 1). ***σ***_1_(*j*) is defined as the *j*th candidate from another (independent) random permutation of the integer set {1,…,*N*}. Finally,
  $$\begin{array}{rl} {\text{G}}_{i} & :=\frac{1}{N}\left\{\begin{array}{c} c({\text{X}}_{i-1}-{\hat{\text{X}}}_{i-1})({{\text{F}}_{i-1}}^{T}\tau_{i}-{{\hat{\text{F}}}_{i-1}}^{T}\tau_{i-1}-\Delta {{\hat{\text{F}}}_{i-1}}^{T}\tau_{i})\\ \;+({\hat{\text{X}}}_{i-1}\tau_{i}-{\hat{\text{X}}}_{i-1}\tau_{i-1}){\left({\text{F}}_{i-1}-{\hat{\text{F}}}_{i-1}\right)}^{T} \end{array}\right\}\\ & \;\times {\left\{\begin{array}{c} c\alpha \frac{1}{N-1}(({\overset{⌢}{\text{F}}}_{i-1}-{\text{F}}_{i-1})-({\overset{⌢}{\text{F}}}_{i-1}-{\hat{\text{F}}}_{i-1}))\\ {\left(({\overset{⌢}{\text{F}}}_{i-1}-{\text{F}}_{i-1})-({\overset{⌢}{\text{F}}}_{i-1}-{\hat{\text{F}}}_{i-1})\right)}^{T}+(1-\alpha )\gamma_{i}\gamma_{i}^{T} \end{array}\right\}}^{-1}. \end{array}$$
4. If $f({\text{x}}_{i}^{\left(j\right)})\geq f({\text{x}}_{i-1}^{\left(j\right)}),\;j=1,\ldots ,N,$ then retain ${\text{x}}_{i}^{\left(j\right)}$ as the updated particle; else set ${\text{x}}_{i}^{\left(j\right)}={\text{x}}_{i-1}^{\left(j\right)}$.
5. If *i*<*M*, go to step 3 with *i*=*i*+1, else terminate the algorithm and report $(1/N)\sum_{j=1}^{N}{\text{x}}_{i}^{\left(j\right)}$ as an estimate for the global optimum.

In the next pseudo-code, the DE-like scalar element-wise scrambling scheme for particles is implemented and the resulting modification accepted with some relaxation criteria. The greedy local search based on the innovation term ${\overset{⌢}{f}}_{i-1}-f_{i-1}^{\left(j\right)}$ is not included in this pseudo-code as it might negatively bear on the global exploration. It may be possible to modify the pseudo-code to augment the innovation vector with additional innovation terms like ${\overset{⌢}{f}}_{i-1}-f_{i-1}^{\left(j\right)}$ after properly weighing the different terms. However, such a variation is presently not attempted.

**COMBEO** *pseudo-code 2*

1. Replicate steps 1 and 2 from *pseudo-code 1*.
2. *Additive update*
  Choose *α*∈(0,1) as in *pseudo-code 1* and update each particle as described below. Draw a random number *r* uniformly from the set {1,…,*n*}. As in *pseudo-code 1*, define *σ*_2_(*j*) as the *j*th candidate from a uniformly random permutation over the integer set {1,…,*N*}.
  Initialize: ${\text{x}}_{i}^{\left(j\right)}={\text{x}}_{i-1}^{\sigma_{2}(j)}$, *j*=1,…,*N*.
  If *ζ*_*i*_<*c*_*i*_ (0<*c*_*i*_≤1 is a scalar chosen by the end-user and *ζ*_*i*_ an independent uniform random variable in [0,1]), then
  $$x_{i}^{r,(j)}=x_{i-1}^{r,\sigma_{2}(j)}+D_{i}^{r,(j)},\;j=1,\ldots ,N,$$
  with ${\text{D}}_{i}^{\left(j\right)}$ as in *pseudo-code 1* and ${\text{I}}_{i}^{\left(j\right)}:=\{{\text{x}}_{i-1}^{\sigma_{1}(j)}-{\text{x}}_{i-1}^{\left(j\right)}\}$, where *σ*_1_(*j*) is a uniformly random permutation on the index set {1,*N*}∖{*j*};
  else
  $$x_{i}^{m,(j)}=x_{i-1}^{m,\sigma_{2}(j)}.$$
  Set *r*→*r*+1. Run the loop on *r* till it reaches *n*.
3. Replicate steps 4 and 5 from *pseudo-code 1*.

By using the available personal and global best information as in the PSO, one may propose yet another global optimization pseudo-code, as suggested earlier while discussing the coalescence step. Thus, the search here is based purely on a martingale problem without requiring a random perturbation, especially the scrambling step. This is an interesting proposal in the sense that the search for the global optimum is attempted by just constructing an innovation vector and driving the same to a zero-mean martingale. In the previous two algorithms (*pseudo-codes 1* and *2*), the update terms were not constructed using past information beyond one step. In *pseudo-code 3* presented below, the update term is however based on the evolution history until the last iteration in order to explore the search space more exhaustively. Without a loss of generality, the specific algorithm given below is for a global maximization problem.

**COMBEO** *pseudo-code 3*

1. Same as in *pseudo-code 1*.
2. Generate the initial population ${\left\{{\text{x}}_{0}^{\left(j\right)}\right\}}_{j=1}^{N}$.
  Initialize: $\vartheta_{\max}=1$ and $\vartheta_{\min}=0.1$ .
  Initialize the local and global best particles ${\;}^{p}{\text{x}}_{0}^{\left(j\right)}={\text{x}}_{0}^{\left(j\right)},\;j=1,\ldots ,N$ and ${\;}^{g}{\text{x}}_{0}=\arg\max\{f({\text{x}}_{0}^{\left(1\right)}),\ldots ,f({\text{x}}_{0}^{\left(N\right)})\}$. Initialize the correction term ${\text{D}}_{0}^{\left(j\right)}=\text{0},\;j=1,\ldots ,N.$ For each discrete *τ*_*i*_, *i*=1,…,*M*−1, execute the following steps.
3. *Additive update*
  Choose *α*∈(0,1).
  Compute $\vartheta_{i}=\vartheta_{\max}-(\vartheta_{\max}-\vartheta_{\min})(i/M)$.
  Update each particle as:
  $${\text{x}}_{i}^{\left(j\right)}={\text{x}}_{i-1}^{\left(j\right)}+{\text{D}}_{i}^{\left(j\right)},\;j=1,\ldots ,N.$$
  ${\text{D}}_{i}^{\left(j\right)}$ is the *j*th update vector. The expression for ${\text{D}}_{i}^{\left(j\right)}$ is also reproduced below:
  $$\begin{array}{rl} {\text{D}}_{i}^{\left(j\right)} & :=\vartheta_{i}{\text{D}}_{i-1}^{\left(j\right)}+\beta_{i}{\text{G}}_{i}{\text{I}}_{i}^{\left(j\right)},\;j=1,\ldots ,N\\ {\tilde{\text{I}}}_{i}^{\left(j\right)} & =\left\{\begin{array}{c} {\tilde{\text{x}}}_{i}^{p(j)}-{\tilde{\text{x}}}_{i}^{\left(j\right)}\\ {\tilde{\text{x}}}_{i}^{g}-{\tilde{\text{x}}}_{i}^{\left(j\right)} \end{array}\right\}. \end{array}$$
  $\beta_{i}={\hat{\beta }}_{i}\zeta_{i}$ (${\hat{\beta }}_{i}$ is a scalar constant and *ζ*_*i*_ a uniform random number in [0,1]).
4. Update the personal and global information as:
  if $f({\text{x}}_{i}^{\left(j\right)})\geq f({\text{x}}_{i-1}^{\left(j\right)}),\;j=1,\ldots ,N,$ then retain ${\;}^{p}{\text{x}}_{i}^{\left(j\right)}={\text{x}}_{i}^{\left(j\right)}$ as the updated particle;
  if $\max\{f({\text{x}}_{i}^{\left(1\right)}),\ldots ,f({\text{x}}_{i}^{\left(N\right)})\}\geq f({\;}^{g}{\text{x}}_{i-1})$ then ${\;}^{g}{\text{x}}_{i}=\arg\max\{f({\text{x}}_{i}^{\left(1\right)}),\ldots ,f({\text{x}}_{i}^{\left(N\right)})\}$;
  else ^*g*^**x**_*i*_=^*g*^**x**_*i*−1_.

Before concluding this section, a word of caution regarding the claims made on a relatively superior performance of the present approach vis-a-vis a few others, e.g. the DE and the PSO, should be in order. Optimization schemes like the DE or the PSO have been extensively improved over the last 15 years or so and it would be wrong to qualify the current method (as captured through the pseudo-codes above) as being the most competent. Nevertheless, the inherent flexibility of our proposal should permit the incorporation of the basic concepts behind some of the recent improvements in the DE or the PSO [30–33], thereby rendering the latter even more effective. Such details however need to be worked out and do not form part of this study.

## 5. Numerical illustrations

### 5.1 Benchmark problems

While the efficiency of a global optimization strategy is largely dependent on its ability to search all the promising search regions, the complexity level of a given optimization problem could exponentially increase with increasing number of design variables. As an example, even though Rosenbrock's function is unimodal in two dimensions (*n*=2), it is highly multimodal in still higher dimensional search spaces. Hence, a global optimization scheme, which works successfully for lower dimensional problems, may very well fail as the dimension of the problem increases. Moreover, the level of difficulty in finding the global optimum may also be dependent on the specific characteristics of the objective function, which goes to explain the varied degree of difficulty in solving different optimization problems of the same dimension. If the problem is separable or partially separable, i.e. if the objective function can be additively split in terms of component functions, each of which is expressible in terms of just one element or a small subset of elements of the design variable vector **x**, the original problem may actually be decoupled into a set of sub-problems. Each sub-problem, involving one scalar variable or a small set of scalar variables, may be solved separately, e.g. by a simple line search for one scalar variable. Depending on the degree of complexity, optimization problems could be categorized as separable, *m*-non-separable and non-separable [34]. In between the two extreme cases, i.e. separable and non-separable, are the *m*-non-separable functionals (*m* being the maximum number of scalar design variables appearing in the descriptions of the component functions) that correspond to partially separable problems. The nomenclature *separability* bears a similar meaning as *epistasis* in biology. In this work, in testing the effectiveness of the proposed schemes to some extent, the following problems have been considered.

I. Separable functions:
  (a) *F*1: shifted elliptic function
  (b) *F*2: shifted Rastrigin's function
  (c) *F*3: shifted Ackley's function
II. Single-group *m*-non-separable functions:
  (d) *F*4: single-group shifted and *m*-rotated elliptic function
  (e) *F*5: single-group shifted and *m*-rotated Rastrigin's function
  (f) *F*6: single-group shifted *m*-dimensional Schwefel's Problem 1.2
  (g) *F*7: single-group shifted *m*-dimensional Rosenbrock's function
III. *n*/2*m*-group *m*-non-separable functions:
  (h) *F*8: *n*/2*m*-group shifted and *m*-rotated Rastrigin's function
  (i) *F*9: *n*/2*m*-group shifted and *m*-rotated Schwefel's Problem 1.2
IV. *n*/*m*-group *m*-non-separable functions:
  (j) *F*10: *n*/*m*-group shifted and *m*-rotated Schwefel's Problem 1.2
V. Non-separable functions:
  (k) *F*11: shifted Schwefel's Problem 1.2

The above functions are constructed using the following basic functions.

*B*1 The sphere function: $F_{\mathrm{sphere}}(\text{x})=\sum_{j=1}^{n}{\left(x^{j}\right)}^{2}$
*B*2 The elliptic function: $F_{\mathrm{elliptic}}(\text{x})=\sum_{j=1}^{n}\left[{\left(10^{6}\right)}^{\left(j-1\right)/\left(n-1\right)}{\left(x^{j}\right)}^{2}\right]$
*B*3 The rotated elliptic function: *F*_rot_elliptic_(**x**)=*F*_elliptic_(**z**), **z**=x*M (**M** being an orthogonal matrix)
*B*4 Schwefel's Problem 1.2: $F_{\mathrm{Schwefel}}(\text{x})=\sum_{k=1}^{n}{\left(\sum_{j=1}^{k}x^{j}\right)}^{2}$
*B*5 Rosenbrock's function: $F_{\mathrm{Rosenbrock}}(\text{x})=\sum_{j=1}^{n-1}\left[100{\left({\left(x^{j}\right)}^{2}-x^{j+1}\right)}^{2}+{\left(x^{j}-1\right)}^{2}\right]$
*B*6 Rastrigin's function: $F_{\mathrm{Rastrigin}}(\text{x})=\sum_{j=1}^{n}\left[{\left(x^{j}\right)}^{2}-10\cos(2\pi x^{j})+10\right]$
*B*7 Rotated Rastrigin's function: *F*_rot_Rastrigin_(**x**)=*F*_Rastrigin_(**z**), **z**=x*M (**M** being a orthogonal matrix)
*B*8 Ackley's function: $F_{\mathrm{Ackley}}(\text{x})=-20\exp\left(-0.2(1/n)\sqrt{\sum_{j=1}^{n}{\left(x^{j}\right)}^{2}}\right)-\exp((1/n)\sum_{j=1}^{n}\cos(2\pi x^{j}))+20+\exp(1)$
*B*9 Rotated Ackley's function: *F*_rot_Ackley_(**x**)=*F*_Ackley_(**z**), **z**=x*M
  Explicit expressions for the functions *F*1–*F*11 are given below. Let **z**=**x**−**o**. $\text{o}\in R^{n}$ denote a shifted global optimum and *σ*_*n*_ the random permutation of the vector {1,…,*n*}.
  (a) *F*1(**x**)=*F*_elliptic_(**z**)
  (b) *F*2(**x**)=*F*_Rastrigin_(**z**)
  (c) *F*3(**x**)=*F*_Ackley_(**z**)
  (d) $F4(\text{x})=F_{\mathrm{rot}\_\mathrm{elliptic}}(\text{z}(\sigma_{n}(1:m)))\times 10^{6}+F_{\mathrm{elliptic}}(\text{z}(\sigma_{n}\left(m+1:n\right)))$
  (e) $F5(\text{x})=F_{\mathrm{rot}\_\mathrm{Rastrigin}}(\text{z}(\sigma_{n}(1:m)))\times 10^{6}+F_{\mathrm{Rastrigin}}(\text{z}(\sigma_{n}\left(m+1:n\right)))$
  (f) $F6(\text{x})=F_{\mathrm{Schwefel}}(\text{z}(\sigma_{n}(1:m)))\times 10^{6}+F_{\mathrm{sphere}}(\text{z}(\sigma_{n}\left(m+1:n\right)))$
  (g) $F7(\text{x})=F_{\mathrm{Rosenbrock}}(\text{z}(\sigma_{n}(1:m)))\times 10^{6}+F_{\mathrm{sphere}}(\text{z}(\sigma_{n}\left(m+1:n\right)))$
  (h) $F8(\text{x})=\sum_{k=1}^{n/2m}\left[\begin{array}{l} F_{\mathrm{rot}\_\mathrm{Rastrigin}}(\text{z}(\sigma_{n}((k-1)m+km)))\times 10^{6}\\ \;+F_{\mathrm{Rastrigin}}\left(\text{z}\left(\sigma_{n}\left(\frac{n}{2}+1:n\right)\right)\right) \end{array}\right]$
  (i) $F9(\text{x})=\sum_{k=1}^{n/2m}\left[\begin{array}{l} F_{\mathrm{Schwefel}}(\text{z}(\sigma_{n}((k-1)m+km)))\times 10^{6}\\ \;+F_{\mathrm{sphere}}\left(\text{z}\left(\sigma_{n}\left(\frac{n}{2}+1:n\right)\right)\right) \end{array}\right]$
  (j) $F10(\text{x})=\sum_{k=1}^{n/m}F_{\mathrm{Schwefel}}(\text{z}(\sigma_{n}((k-1)m+km)))$
  (k) *F*11(**x**)=*F*_Schwefel_(**z**).

In solving optimization problems involving functions *F*1–*F*11, the performance of *pseudo-code 2*, as compared with the parent DE, is given in table 1. The population set in both the cases consists of 2000 particles. The scalar parameter *c*_*i*_, which is similar to the acceptance criteria given by the crossover constant (CR) in DE [3] is taken as 0.1. In implementing DE, CR is also taken as 0.1. For both the schemes, the programme is terminated if the number of iterations exceeds the maximum allowed threshold, which is presently given by it_max=1×10^5^. In all the tables, *ε* denotes an error norm ≤10^−5^. By the way of investigating the consistent reproducibility of the results reported herein, the proposed optimization scheme (*pseudo-code 2*) and the DE are run five times for *F*3. It is observed that in all the cases *pseudo-code 2* provides the optimum in roughly the same number of iterations (same range) mentioned in table 2, whereas the DE fails to converge in all the runs. In the literature, the above benchmark problems are typically considered to be ‘difficult’ to solve by most of the existing evolutionary optimization schemes [34].

**Table 1.** Comparative performance of *pseudo-code 2* and DE against objective functions *F*1–*F*11 (*n*=40). (OF, objective functional; NI, number of iterations; MEN, mean error norm.)

	*pseudo-code 2*		DE	
OF	NI	MEN	NI	MEN
*F*1	1.69×10^3^	*ε*	2.42×10^4^	*ε*
*F*2	4.68×10^3^	*ε*	2.16×10^4^	*ε*
*F*3	3.73×10^3^	*ε*	$it_{\max}$	20
*F*4	1.34×10^4^	*ε*	$it_{\max}$	1.2×10^4^
*F*5	3×10^3^	*ε*	2.37×10^4^	*ε*
*F*6	2.51×10^3^	*ε*	2.12×10^4^	*ε*
*F*7	1.11×10^4^	*ε*	2.81×10^4^	*ε*
*F*8	4.15×10^4^	*ε*	5.84×10^4^	*ε*
*F*9	2.29×10^3^	*ε*	3.62×10^4^	*ε*
*F*10	8.84×10^3^	*ε*	it_max	575
*F*11	it_max	7.7×10^−4^	it_max	5.78×10^3^

**Table 2.** Comparative performance of *pseudo-code 3*, *pseudo-code 1* and PSO against objective functions *B*1–*B*9 (*n*=40). (OF, objective functional; NI, number of iterations; MEN, mean error norm.)

	*pseudo-code 3 (pseudo-code 1)*		PSO	
OF	NI	MEN	NI	MEN
*B*1	146 (37)	*ε*	330	*ε*
*B*2	172 (55)	*ε*	353	*ε*
*B*3	212 (64)	*ε*	630	*ε*
*B*4	190 (49)	*ε*	456	*ε*
*B*5	154 (35)	*ε*	280	*ε*
*B*6	155 (42)	*ε*	353	*ε*
*B*7	172 (46)	*ε*	330	*ε*
*B*8	195 (61)	*ε*	485	*ε*
*B*9	201 (59)	*ε*	440	*ε*

Indeed, for these problems, the PSO, *pseudo-codes 1* and *3* could not converge to the global optimum within the presently allowed number of iterations. However, in order to demonstrate that *pseudo-codes 1 and 3* could indeed be very accurate with faster convergence for many global optimization problems, these codes are tested with benchmark global optimization problems *B*1–*B*9. While these problems are also challenging in their own right and often used in the literature to assess the performance of optimization algorithms, they do pose a comparatively lesser degree of complexity in reaching the global optimum vis-a-vis *F*1–*F*11. The results, reported in table 2, are also compared against the PSO under a similar environment. In all the cases, the population set is taken to consist of 50 particles. As anticipated, *pseudo-code 3* and especially *pseudo-code 1* show conspicuously faster convergence than the PSO while maintaining the same or higher level of accuracy. In any case, there is clearly scope for improving these two pseudo-codes (*1* and *3*) so that they can be rendered as competitive as *pseudo-code 2*. In exploring modification over the present algorithms so as to render them more robust and applicable to higher dimensional optimization problems, one possible way, for instance, would be *intelligent* augmentation of multiple adaptive strategies [35]. Moreover, since there are no specific formulae for selecting the parameter values in the present algorithms, parameter control mechanisms within the martingale problem set-up may also have to be suitably devised [35].

In order to gain further understanding on the performance of COMBEO, *pseudo-code* 2 is tested against yet another set of benchmark problems with the cost functionals, herein denoted as *F*(*I*)1–24, as considered in (http://coco.gforge.inria.fr/doku.php). In evolutionary algorithms, tuning of some of the parameters could be a tricky issue [36,37]. Since the performance of a scheme may crucially depend upon the choice of such parameters [36,37], which are often left to be selected at the user-end, a robust scheme should have the number of such tunable parameters kept minimum. Note that only one tuning parameter (CR) is introduced in the proposed scheme (*pseudo-code 2*). A detailed comparison of the performance of CMA-ES vis-a-vis COMBEO with varying CR values (e.g. 0.1, 0.9 and a number randomly drawn from a uniform distribution over 0 and 1) in the context of the new set of benchmark problems as considered in (http://coco.gforge.inria.fr/doku.php) has been undertaken and the results are reported in tables 3 and 4, only for the case of *n*=20. In order to limit the length of the article, a more detailed comparison for different values of *n* is provided in the electronic supplementary material. It may be noted that, in order to maintain a fair comparison between COMBEO and CMA-ES, the realized state with the minimum cost functional value in the available ensemble is considered as the solution. In these tables, EFE, error 1, error 2, error 3 and error 4, respectively, denote the average number of function evaluations among independent restarts, mean error, best error, worst error and median error. Tables 3 and 4 and electronic supplementary material, tables SI–SX, also include simulation results via CMA-ES [6]. The source code for CMA-ES is taken from https://www.lri.fr/~hansen/cmaes_inmatlab.html.

**Table 3.** Comparative performance of *pseudo-code 2* (with varying CR) and CMAES against objective functions *F*(*I*)1–*F*(*I*)14 (*n*=20).

*F*(*I*) index	method	*EF(I)E*	error 1	error 2	error 3	error 4
*F*(*I*)1	*pseudo-code 2*(CR=0.1)	32 909	8.70×10^−9^	8.70×10^−9^	8.70×10^−9^	8.70×10^−9^
*F*(*I*)1	*pseudo-code 2*(CR=rand)	50 909	7.98×10^−9^	7.98×10^−9^	7.98×10^−9^	7.98×10^−9^
*F*(*I*)1	*pseudo-code 2*(CR=0.9)	1 000 000	4.95×10^−5^	3.24×10^−5^	5.98×10^−5^	4.44×10^−5^
*F*(*I*)1	CMA-ES	4537	−1.00×10^−8^	−1.00×10^−8^	−1.00×10^−8^	−1.00×10^−8^
*F*(*I*)2	*pseudo-code 2*(CR=0.1)	46 818	8.31×10^−9^	8.31×10^−9^	8.31×10^−9^	8.31×10^−9^
*F*(*I*)2	*pseudo-code 2*(CR=rand)	70 455	6.67×10^−9^	6.67×10^−9^	6.67×10^−9^	6.67×10^−9^
*F*(*I*)2	*pseudo-code 2*(CR=0.9)	1 000 000	4.15×10^−1^	3.30×10^−1^	6.39×10^−1^	4.09×10^−1^
*F*(*I*)2	CMA-ES	21 147	−1.00×10^−8^	−1.00×10^−8^	−1.00×10^−8^	−1.00×10^−8^
*F*(*I*)3	*pseudo-code 2*(CR=0.1)	72 909	9.50×10^−9^	9.50×10^−9^	9.50×10^−9^	9.50×10^−9^
*F*(*I*)3	*pseudo-code 2*(CR=rand)	1 000 000	5.68×10^0^	5.32×10^0^	9.32×10^0^	5.32×10^0^
*F*(*I*)3	*pseudo-code 2*(CR=0.9)	1 000 000	9.49×10^1^	8.92×10^1^	1.07×10^2^	9.71×10^1^
*F*(*I*)3	CMA-ES	7822	2.33×10^1^	1.99×10^1^	2.59×10^1^	2.39×10^1^
*F*(*I*)4	*pseudo-code 2*(CR=0.1)	79 727	6.87×10^−9^	6.87×10^−9^	6.87×10^−9^	6.87×10^−9^
*F*(*I*)4	*pseudo-code 2*(CR=rand)	1 000 000	9.32×10^0^	8.89×10^0^	1.20×10^1^	8.89×10^0^
*F*(*I*)4	*pseudo-code 2*(CR=0.9)	1 000 000	1.04×10^2^	1.01×10^2^	1.06×10^2^	1.06×10^2^
*F*(*I*)4	CMA-ES	7421	3.62×10^1^	2.69×10^1^	5.27×10^1^	2.89×10^1^
*F*(*I*)5	*pseudo-code 2*(CR=0.1)	4727	−1.00×10^−8^	−1.00×10^−8^	−1.00×10^−8^	−1.00×10^−8^
*F*(*I*)5	*pseudo-code 2*(CR=rand)	3455	−1.00×10^−8^	−1.00×10^−8^	−1.00×10^−8^	−1.00×10^−8^
*F*(*I*)5	*pseudo-code 2*(CR=0.9)	2818	−1.00×10^−8^	−1.00×10^−8^	−1.00×10^−8^	−1.00×10^−8^
*F*(*I*)5	CMA-ES	340	−1.00×10^−8^	−1.00×10^−8^	−1.00×10^−8^	−1.00×10^−8^
*F*(*I*)6	*pseudo-code 2*(CR=0.1)	1 000 000	1.49×10^−2^	1.42×10^−2^	2.08×10^−2^	1.42×10^−2^
*F*(*I*)6	*pseudo-code 2*(CR=rand)	1 000 000	2.06×10^−1^	1.58×10^−1^	2.65×10^−1^	1.58×10^−1^
*F*(*I*)6	*pseudo-code 2*(CR=0.9)	1 000 000	5.54×10^2^	4.62×10^2^	6.44×10^2^	5.65×10^2^
*F*(*I*)6	CMA-ES	16 332	−1.00×10^−8^	−1.00×10^−8^	−1.00×10^−8^	−1.00×10^−8^
*F*(*I*)7	*pseudo-code 2*(CR=0.1)	1 000 000	1.15×10^0^	1.05×10^0^	1.50×10^0^	1.11×10^0^
*F*(*I*)7	*pseudo-code 2*(CR=rand)	1 000 000	1.46×10^0^	1.05×10^0^	1.82×10^0^	1.74×10^0^
*F*(*I*)7	*pseudo-code 2*(CR=0.9)	1 000 000	3.97×10^1^	3.81×10^1^	4.02×10^1^	4.02×10^1^
*F*(*I*)7	CMA-ES	3126	4.99×10^0^	4.07×10^0^	6.84×10^0^	4.68×10^0^
*F*(*I*)8	*pseudo-code 2*(CR=0.1)	1 000 000	1.20×10^1^	1.17×10^1^	1.26×10^1^	1.17×10^1^
*F*(*I*)8	*pseudo-code 2*(CR=rand)	1 000 000	1.52×10^1^	1.51×10^1^	1.56×10^1^	1.51×10^1^
*F*(*I*)8	*pseudo-code 2*(CR=0.9)	1 000 000	1.92×10^1^	1.88×10^1^	2.00×10^1^	1.92×10^1^
*F*(*I*)8	CMA-ES	21 876	−1.00×10^−8^	−1.00×10^−8^	−1.00×10^−8^	−1.00×10^−8^
*F*(*I*)9	*pseudo-code 2*(CR=0.1)	1 000 000	1.73×10^1^	1.72×10^1^	1.73×10^1^	1.73×10^1^
*F*(*I*)9	*pseudo-code 2*(CR=rand)	1 000 000	1.66×10^1^	1.65×10^1^	1.67×10^1^	1.67×10^1^
*F*(*I*)9	*pseudo-code 2*(CR=0.9)	1 000 000	1.83×10^1^	1.81×10^1^	1.85×10^1^	1.84×10^1^
*F*(*I*)9	CMA-ES	22 236	−1.00×10^−8^	−1.00×10^−8^	−1.00×10^−8^	−1.00×10^−8^
*F*(*I*)10	*pseudo-code 2*(CR=0.1)	1 000 000	2.83×10^4^	2.65×10^4^	3.14×10^4^	2.65×10^4^
*F*(*I*)10	*pseudo-code 2*(CR=rand)	1 000 000	1.01×10^5^	8.36×10^4^	1.55×10^5^	8.36×10^4^
*F*(*I*)10	*pseudo-code 2*(CR=0.9)	1 000 000	3.79×10^5^	3.53×10^5^	6.37×10^5^	3.53×10^5^
*F*(*I*)10	CMA-ES	21 391	−1.00×10^−8^	−1.00×10^−8^	−1.00×10^−8^	−1.00×10^−8^
*F*(*I*)11	*pseudo-code 2*(CR=0.1)	1 000 000	6.29×10^1^	6.29×10^1^	6.29×10^1^	6.29×10^1^
*F*(*I*)11	*pseudo-code 2*(CR=rand)	1 000 000	1.69×10^2^	1.44×10^2^	2.19×10^2^	1.60×10^2^
*F*(*I*)11	*pseudo-code 2*(CR=0.9)	1 000 000	1.31×10^2^	1.15×10^2^	2.23×10^2^	1.15×10^2^
*F*(*I*)11	CMA-ES	17 781	−1.00×10^−8^	−1.00×10^−8^	−1.00×10^−8^	−1.00×10^−8^
*F*(*I*)12	*pseudo-code 2*(CR=0.1)	1 000 000	1.13×10^−1^	2.05×10^−2^	1.33×10^−1^	1.33×10^−1^
*F*(*I*)12	*pseudo-code 2*(CR=rand)	1 000 000	9.25×10^−2^	1.52×10^−2^	3.05×10^−1^	1.52×10^−2^
*F*(*I*)12	*pseudo-code 2*(CR=0.9)	1 000 000	1.72×10^3^	1.42×10^3^	3.27×10^3^	1.42×10^3^
*F*(*I*)12	CMA-ES	36 406	−1.00×10^−8^	−1.00×10^−8^	−1.00×10^−8^	−1.00×10^−8^
*F*(*I*)13	*pseudo-code 2*(CR=0.1)	1 000 000	5.08×10^−1^	1.60×10^−1^	1.31×10^0^	4.58×10^−1^
*F*(*I*)13	*pseudo-code 2*(CR=rand)	1 000 000	5.11×10^−2^	3.30×10^−2^	8.28×10^−2^	3.30×10^−2^
*F*(*I*)13	*pseudo-code 2*(CR=0.9)	1 000 000	4.38×10^1^	4.31×10^1^	4.38×10^1^	4.38×10^1^
*F*(*I*)13	CMA-ES	8332	2.96×10^−7^	2.96×10^−7^	2.96×10^−7^	2.96×10^−7^
*F*(*I*)14	*pseudo-code 2*(CR=0.1)	1 000 000	2.91×10^−3^	2.46×10^−3^	4.67×10^−3^	2.46×10^−3^
*F*(*I*)14	*pseudo-code 2*(CR=rand)	1 000 000	5.81×10^−3^	4.56×10^−3^	8.44×10^−3^	4.56×10^−3^
*F*(*I*)14	*pseudo-code 2*(CR=0.9)	1 000 000	8.22×10^−2^	8.22×10^−2^	8.22×10^−2^	8.22×10^−2^
*F*(*I*)14	CMA-ES	36 078	−1.00×10^−8^	−1.00×10^−8^	−1.00×10^−8^	−1.00×10^−8^

**Table 4.** Comparative performance of *pseudo-code 2* (with varying CR) and CMAES against objective functions *F*(*I*)1–*F*(*I*)14 (*n*=20).

*F*(*I*) index	method	*EF(I)E*	error 1	error 2	error 3	error 4
*F*(*I*)15	*pseudo-code 2*(CR=0.1)	1 000 000	8.66×10^1^	8.11×10^1^	9.36×10^1^	8.54×10^1^
*F*(*I*)15	*pseudo-code 2*(CR=rand)	1 000 000	8.94×10^1^	8.48×10^1^	1.05×10^2^	8.48×10^1^
*F*(*I*)15	*pseudo-code 2*(CR=0.9)	1 000 000	1.22×10^2^	1.21×10^2^	1.33×10^2^	1.21×10^2^
*F*(*I*)15	CMA-ES	7657	1.69×10^1^	1.69×10^1^	1.69×10^1^	1.69×10^1^
*F*(*I*)16	*pseudo-code 2*(CR=0.1)	1 000 000	1.40×10^1^	1.09×10^1^	1.64×10^1^	1.41×10^1^
*F*(*I*)16	*pseudo-code 2*(CR=rand)	1 000 000	1.55×10^1^	1.52×10^1^	1.71×10^1^	1.52×10^1^
*F*(*I*)16	*pseudo-code 2*(CR=0.9)	1 000 000	1.94×10^1^	1.83×10^1^	2.20×10^1^	1.83×10^1^
*F*(*I*)16	CMA-ES	11 041	7.93×10^−1^	6.31×10^−1^	2.41×10^0^	6.31×10^−1^
*F*(*I*)17	*pseudo-code 2*(CR=0.1)	1 000 000	1.18×10^−2^	8.39×10^−3^	1.70×10^−2^	1.20×10^−2^
*F*(*I*)17	*pseudo-code 2*(CR=rand)	1 000 000	9.43×10^−3^	8.35×10^−3^	1.34×10^−2^	8.35×10^−3^
*F*(*I*)17	*pseudo-code 2*(CR=0.9)	1 000 000	5.46×10^−1^	5.03×10^−1^	6.17×10^−1^	5.44×10^−1^
*F*(*I*)17	CMA-ES	12 505	7.83×10^−3^	3.43×10^−3^	1.40×10^−2^	4.95×10^−3^
*F*(*I*)18	*pseudo-code 2*(CR=0.1)	1 000 000	3.45×10^−1^	2.93×10^−1^	4.10×10^−1^	3.39×10^−1^
*F*(*I*)18	*pseudo-code 2*(CR=rand)	1 000 000	1.65×10^−1^	1.50×10^−1^	1.90×10^−1^	1.50×10^−1^
*F*(*I*)18	*pseudo-code 2*(CR=0.9)	1 000 000	3.22×10^0^	3.18×10^0^	3.59×10^0^	3.18×10^0^
*F*(*I*)18	CMA-ES	12 964	4.95×10^−2^	4.12×10^−2^	7.00×10^−2^	4.12×10^−2^
*F*(*I*)19	*pseudo-code 2*(CR=0.1)	1 000 000	3.07×10^0^	2.75×10^0^	3.30×10^0^	3.12×10^0^
*F*(*I*)19	*pseudo-code 2*(CR=rand)	1 000 000	2.65×10^0^	2.53×10^0^	3.89×10^0^	2.53×10^0^
*F*(*I*)19	*pseudo-code 2*(CR=0.9)	1 000 000	4.29×10^0^	4.02×10^0^	4.95×10^0^	4.02×10^0^
*F*(*I*)19	CMA-ES	147 687	1.62×10^0^	7.59×10^−1^	4.16×10^0^	7.59×10^−1^
*F*(*I*)20	*pseudo-code 2*(CR=0.1)	1 000 000	5.07×10^−2^	4.96×10^−2^	6.16×10^−2^	4.96×10^−2^
*F*(*I*)20	*pseudo-code 2*(CR=rand)	1 000 000	6.86×10^−1^	6.43×10^−1^	6.98×10^−1^	6.98×10^−1^
*F*(*I*)20	*pseudo-code 2*(CR=0.9)	1 000 000	2.41×10^0^	2.38×10^0^	2.44×10^0^	2.44×10^0^
*F*(*I*)20	CMA-ES	5963	1.57×10^0^	1.39×10^0^	1.81×10^0^	1.55×10^0^
*F*(*I*)21	*pseudo-code 2*(CR=0.1)	1 000 000	1.31×10^−2^	9.34×10^−4^	1.44×10^−2^	1.44×10^−2^
*F*(*I*)21	*pseudo-code 2*(CR=rand)	87 364	9.12×10^−9^	9.12×10^−9^	9.12×10^−9^	9.12×10^−9^
*F*(*I*)21	*pseudo-code 2*(CR=0.9)	1 000 000	2.07×10^0^	1.52×10^0^	2.54×10^0^	2.51×10^0^
*F*(*I*)21	CMA-ES	5113	7.29×10^0^	6.92×10^−1^	6.44×10^1^	1.80×10^0^
*F*(*I*)22	*pseudo-code 2*(CR=0.1)	1 000 000	1.95×10^0^	1.95×10^0^	1.95×10^0^	1.95×10^0^
*F*(*I*)22	*pseudo-code 2*(CR=rand)	1 000 000	1.95×10^0^	1.95×10^0^	1.95×10^0^	1.95×10^0^
*F*(*I*)22	*pseudo-code 2*(CR=0.9)	1 000 000	1.96×10^0^	1.96×10^0^	1.96×10^0^	1.96×10^0^
*F*(*I*)22	CMA-ES	4753	2.52×10^0^	6.92×10^−1^	1.83×10^1^	6.92×10^−1^
*F*(*I*)23	*pseudo-code 2*(CR=0.1)	1 000 000	1.41×10^0^	1.12×10^0^	1.58×10^0^	1.58×10^0^
*F*(*I*)23	*pseudo-code 2*(CR=rand)	1 000 000	1.28×10^0^	1.16×10^0^	1.83×10^0^	1.16×10^0^
*F*(*I*)23	*pseudo-code 2*(CR=0.9)	1 000 000	1.88×10^0^	1.73×10^0^	2.34×10^0^	1.73×10^0^
*F*(*I*)23	CMA-ES	3823	2.90×10^0^	2.64×10^0^	3.15×10^0^	3.04×10^0^
*F*(*I*)24	*pseudo-code 2*(CR=0.1)	1 000 000	1.05×10^2^	1.03×10^2^	1.12×10^2^	1.04×10^2^
*F*(*I*)24	*pseudo-code 2*(CR=rand)	1 000 000	9.77×10^1^	9.27×10^1^	1.12×10^2^	9.27×10^1^
*F*(*I*)24	*pseudo-code 2*(CR=0.9)	1 000 000	1.30×10^2^	1.25×10^2^	1.36×10^2^	1.30×10^2^
*F*(*I*)24	CMA-ES	6175	4.67×10^1^	3.89×10^1^	4.96×10^1^	4.96×10^1^

As we see from tables 3 and 4 and the ones included in the electronic supplementary material, while COMBEO generally scores in achieving higher accuracy while approaching the global optimum, CMA-ES does have a distinct computational advantage in that it can perform with substantively lower ensemble sizes, *N*. It would thus be worthwhile to incorporate some of the adaptive features of CMA-ES within COMBEO in a future work.

### 5.2 An application to quantitative estimation of density variation in high-speed flows through inversion of light travel-time data

Having assessed the proposed global optimization framework through a variety of benchmark problems, we now consider a considerably larger dimensional optimization problem that involves the quantitative estimation of density variation in a high-speed flow obstructed by a blunt-nose aerodynamic vehicle. This example is taken up to demonstrate that, while *pseudo-code 2* often provides an effective search tool for the global extremum of complex objective functionals, a greedier search scheme (as in *pseudo-code 1* or its possible variants) could nevertheless be more appropriate for many practically useful, yet large dimensional, problems where a scheme like *pseudo-code 2* (or most available global schemes such as the DE or the PSO) might prove computationally prohibitive.

**Figure 1.**
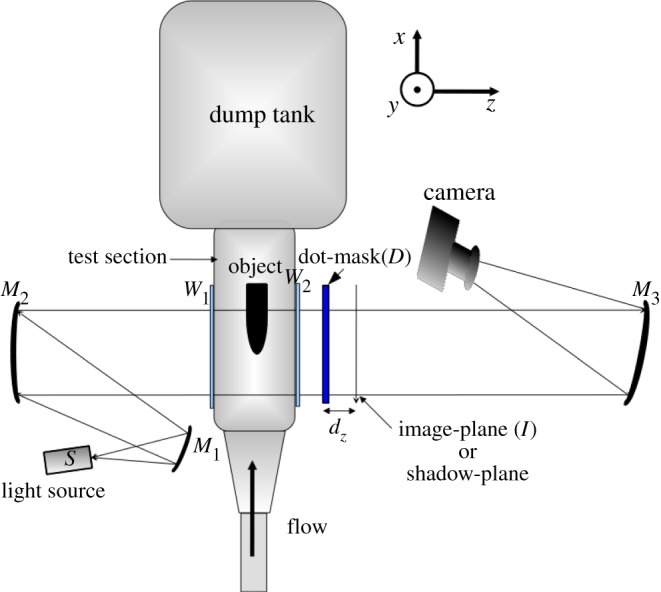
A schematic diagram of the experimental set-up.

**Figure 2.**
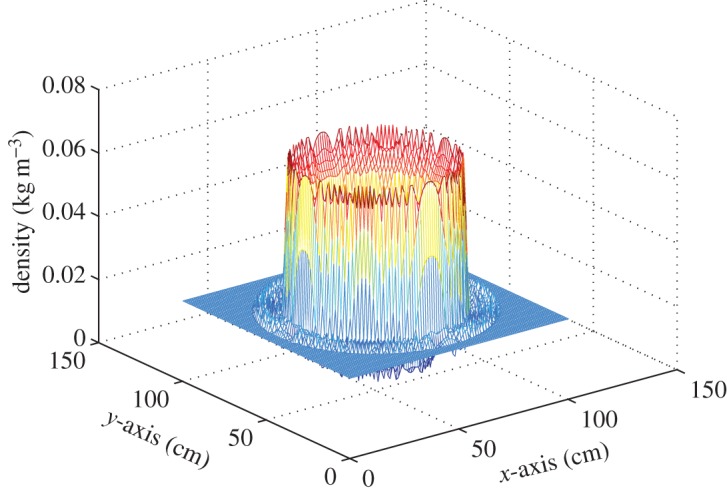
A three-dimensional view of the reconstruction via the proposed framework.

The experimental set-up is shown in figure 1, see [38] for more details. Here, the region of interest (ROI), the flow around the object (in this case, a blunt-nosed missile model), is illuminated by a plane wave. The distorted plane wave trans-illuminates a random dot pattern (RDP). The RDP in effect serves the purpose of the expensive lenslet split Shack–Hartmann sensor. The geometric shadow cast at an adjacent plane is imaged by a high-speed camera. The shadow, in comparison to the original RDP, is space-shifted. Local mean shifts ***d***=(*d*_*x*_,*d*_*y*_) and a related quantity, the slopes of the wavefront ***θ*=**(*d*_*x*_/*d*_*z*_,*d*_*y*_/*d*_*z*_), are estimated by cross-correlating local sub-region of the distorted RDP with the corresponding original and finding the shift of the cross-correlation peak. The estimated local slopes are integrated to find the smooth wavefront *Φ* [39]. The recovered unwrapped *Φ*, which carries the distortion owing to its passage through the ROI, is shown in figure 2. The distorted wavefront *Φ*^*i*^ (or equivalently the phase delay) evaluated at the *i*th detector, is related to the refractive index distribution *ϱ*(**r**) in the ROI, **r**=(*x*,*y*,*z*) being the position vector, through the equation:

$$\Phi^{i}=\frac{2\pi }{\lambda }\int_{0}^{L^{i}}\varrho (\text{r})\;ds.$$

Here *L*^*i*^ is the length of the *i*th ray and ds is an element from the Fermat's path of the light-ray through *ϱ*(**r**), which obeys the Eikonel equation:

$$\frac{d}{ds}\left(\varrho \frac{d\text{r}}{ds}\right)=\nabla \varrho .$$

Therefore, the equation connecting *Φ*^*i*^ to *ϱ*(**r**) is nonlinear. The collection of *Φ*^*i*^ at all the detectors is denoted using the vector ***Φ***:={*Φ*^*i*^}. The problem of recovery of *ϱ*(**r**) from the experimentally measured data, denoted by ***Φ***_exp_, obtained from *Φ* corresponding to the locations of detectors, is posed as an optimization problem which is solved within the proposed framework. The corresponding objective functional may be written as

$$f_{\exp}:=\sqrt{{\left(\Phi_{\exp}-\Phi \right)}^{T}(\Phi_{\exp}-\Phi )}.$$

However, given the large system dimension (*n*=101^2^) and with the algorithm requiring repetitive numerical inversions for *Φ*, most available global optimization schemes (e.g. the DE or the PSO) are rendered impracticable using the commonly available computing systems. An acceleration of the computing speed is however possible in the present framework by so modifying the innovation processes as to render the search greedier while retaining some features of the global search, especially the scrambling step. One way could be to start with *pseudo-code 1*. In order to make the algorithm faster, we might replace the innovation used in *pseudo-code 1* by the difference of the objective functional *f*_exp_ computed over two succeeding iterations. Unfortunately, this leads to instability in the algorithm owing to the fact that the innovation becomes one-dimensional in contrast to the number of design variables which is of order *O*(10^4^). This may be remedied by splitting the original objective functional into 101^2^ individual ones (i.e. one corresponding to each design variable) and thus constructing 101^2^ separate scalar innovation processes for the new objective function vector. This enables solving the problem in only a few iterations (as few as just five iterations) with only 30 MC particles. Accordingly, we now redefine $f_{\exp}^{2}=\sum_{\varsigma =1}^{n}g_{\exp}^{\varsigma }$, where $g_{\exp}^{\varsigma }:={\left(\Phi_{\exp}^{\varsigma }-\Phi^{\varsigma }\right)}^{2}$. The present modification requires the realized innovation **I**_*i*_ at the *i*th iteration in *pseudo-code 1* (specifically in step 3) to be given as

$${\text{I}}_{i}:=\left\{\begin{array}{c} {g_{\exp}^{1}}_{i-1}\\ \cdots \\ {g_{\exp}^{n}}_{i-1} \end{array}\right\}.$$

It is recalled that this modified algorithm uses only scrambling for effecting the global search. This is an interesting example of the practical advantages in being able to suitably design the innovation process within the present framework. It is worth noting that our efforts at rendering the DE and the PSO greedier by tuning their appropriate algorithmic parameters have failed to make them effective in solving the current problem.

**Figure 3.**
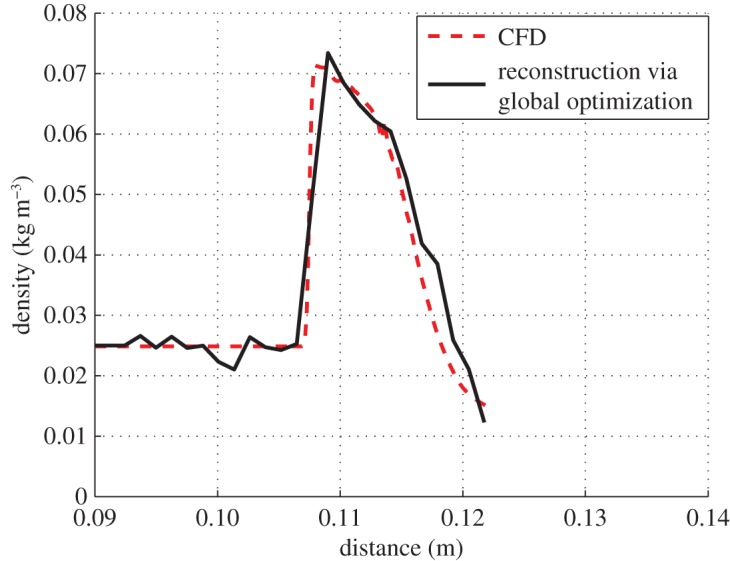
A projection of the reconstruction via the proposed framework.

The recovered *ϱ***(r)** is converted into density distribution using the Gladstone–Dale equation. A cross section of the recovered density profile is shown in figures 2 and 3. The above reconstruction is verified (figure 3) by computing the flow density distribution close to the boundary layer by solving Navier–Stoke's equation using the commercially available CFD software Fluent. The comparison of the density variations near the boundary layer, shown in figure 3, is seen to be quite close.

## 6. Concluding remarks

Despite the widely reported success and appealing simplicity of the methodological aspects of many existing evolutionary schemes for global optimization, often inspired by biological or social observations, the specific forms of their update scheme in approaching the global extremum are generally not rigorously grounded. While a major aspect of this work is the attempt at finding an underlying logical thread that perhaps relates the basic ideas behind many such update strategies, the notion of a non-uniquely specifiable martingale approach in deriving the update term and its functional similarity with the directional derivative, used in most deterministic optimization schemes, has by far been the most fundamental aspect in this article. Such a directional search, free from the notion of classical derivatives, has indeed been responsible for superior convergence features of the COMBEO family of schemes in contrast to some of the well-known evolutionary methods, e.g. the PSO, the DE and the CMA-ES. An effective global exploration of the search space however requires several random perturbation strategies, of which the coalescence step is treated as part of the martingale problem. It is shown that a variant of the coalescence step may mimic a PSO-like search. A DE-like search, on the other hand, may be organized through a variant of scrambling, which is yet another random perturbation strategy involving directional information swaps. The framework of COMBEO, founded on the theory of stochastic processes, promises to yield highly competitive update schemes in order to meet the conflicting demands of faster convergence and efficacious exploration. As demonstrated via numerical work on a host of higher dimensional benchmark objective functions, the COMBEO schemes show consistently superior performance in comparison to several existing evolutionary methods, prominently the DE and the PSO. The flexibility of the proposed optimization paradigm is also demonstrated by evolving a greedier version of the search scheme and applying the same to the reconstruction of boundary layer density variation in a high-speed flow experiment.

The generality of the framework afforded by COMBEO should motivate further research in improving the specific numerical schemes considered in this work. For instance, the COMBEO algorithms could possibly be rendered computationally more competitive by incorporating the adaptive features of the CMA-ES or some of the many available modifications or augmentations of the DE.

## Supplementary Material

The supplementary material (Supplementay_material.tex) contains a detailed comparison, in tabular forms, of the proposed algorithm with CMA-ES.

## Data accessibility

Parts of the data supporting the claims in the article (i.e. those not provided in the main text) have been included in the electronic supplementary material. The results reported therein have been generated using the resources available in the following links: (i) https://www.lri.fr/hansen/cmaes.inmatlab.html. (ii) http://coco.gforge.inria.fr/doku.php.

## A. Appendix A. Derivation of the extremal equation

Theorem A.1 *For* $\phi \in C_{b}^{2}$ *the normalized conditional law π*_*τ*_*(ϕ) of the solution process* **x**_*τ*_ *satisfies the following extremal equation:
*

$$d\pi_{\tau }(\phi )=\frac{1}{2}\pi_{\tau }\left(\sum_{j,k=1}^{n}\sum_{l=1}^{d}\left(\frac{\partial^{2}\phi }{\partial x^{j}\partial x^{k}}\right)g^{jl}g^{kl}\right)d\tau +(\pi_{\tau }(\phi h)-\pi_{\tau }(\phi )\pi_{\tau }(h))(d{\overset{⌣}{f}}_{\tau }-\pi_{\tau }(h)\;d\tau ).$$

Proof. The gain based update equation for a bounded and at least twice continuously differentiable function *ϕ*_*τ*_:=*ϕ*(**x**_*τ*_) of **x**_*τ*_ may be arrived at by expanding *ϕ*(**x**_*τ*_)*Λ*_*τ*_, where *τ*∈(*τ*_*i*−1_,*τ*_*i*_], using Ito's formula:

A 1 $$d(\phi_{\tau }\Lambda_{\tau })=\phi_{\tau }\;d\Lambda_{\tau }+d\phi_{\tau }\Lambda_{\tau }+\langle d\phi_{\tau },\;d\Lambda_{\tau }\rangle ,$$

〈⋅〉 denotes the quadratic covariation. A further expansion leads to

A 2 $$d(\phi_{\tau }\Lambda_{\tau })=\phi_{\tau }\Lambda_{\tau }h_{\tau }\;d{\overset{⌣}{f}}_{\tau }+\Lambda_{\tau }{\phi^{\prime }}_{\tau }^{T}\;d{\text{x}}_{\tau }+\frac{1}{2}\Lambda_{\tau }\langle d{\text{x}}_{\tau },{\phi^{\prime \prime }}_{\tau }\;d{\text{x}}_{\tau }\rangle .$$

By explicitly writing out the term 〈*d***x**_*τ*_,*ϕ*′′_*τ*_ *d***x**_*τ*_〉, we get

A 3 $$d(\phi_{\tau }\Lambda_{\tau })=\phi_{\tau }\Lambda_{\tau }h_{\tau }\;d{\overset{⌣}{f}}_{\tau }+\Lambda_{\tau }{{\phi^{\prime }}_{\tau }}^{T}\;d{\text{x}}_{\tau }+\frac{1}{2}\sum_{j,k=1}^{n}\sum_{l=1}^{d}{\left(\frac{\partial^{2}\phi }{\partial x^{j}\partial x^{k}}\right)}_{\tau }g^{jl}g^{kl}\;d\tau .$$

 ▪

In deriving the above equation, equation (2.1) is made use of. The incremental form in equation (A 3) may be given the following integral representation:

A 4 $$\phi_{\tau }\Lambda_{\tau }=\phi_{i-1}\Lambda_{i-1}+\int_{\tau_{i-1}}^{\tau }\Lambda_{s}\phi_{s}h_{s}d{\overset{⌣}{f}}_{s}+\int_{\tau_{i-1}}^{\tau }\Lambda_{s}\left({\phi^{\prime }}_{s}\;d\xi_{s}+\frac{1}{2}\sum_{j,k=1}^{n}\sum_{l=1}^{d}{\left(\frac{\partial^{2}\phi }{\partial x^{j}\partial x^{k}}\right)}_{s}g^{jl}g^{kl}\right)ds.$$

Taking conditional expectation with respect to $N_{\tau }$ under *Q*, we get

A 5 $$\begin{array}{rl} E_{Q}(\phi_{\tau }\Lambda_{\tau }|N_{\tau }) & =E_{Q}(\phi_{i-1}\Lambda_{i-1}|N_{\tau })+E_{Q}\left((\int_{\tau_{i-1}}^{\tau }\Lambda_{s}\phi_{s}h_{s}\;d⌣f_{s})\right|N_{\tau })+E_{Q}\left((\int_{\tau_{i-1}}^{\tau }\Lambda_{s}{\phi^{\prime }}_{s}\;d\xi_{s})\right|N_{\tau })\\ & \;+\frac{1}{2}E_{Q}\left(\left(\int_{\tau_{i-1}}^{\tau }\sum_{j,k=1}^{n}\sum_{l=1}^{d}{\left(\frac{\partial^{2}\phi }{\partial x^{j}\partial x^{k}}\right)}_{s}g^{jl}g^{kl}\;ds)\right|N_{\tau }\right). \end{array}$$

Using Fubini's theorem,

A 6 $$\begin{array}{rl} E_{Q}(\phi_{\tau }\Lambda_{\tau }|N_{\tau }) & =E_{Q}(\phi_{i-1}\Lambda_{i-1}|N_{\tau })+\int_{\tau_{i-1}}^{\tau }E_{Q}(\Lambda_{s}\phi_{s}h_{s}|N_{s})\;d{\overset{⌣}{f}}_{s}\\ & \;+\int_{\tau_{i-1}}^{\tau }E_{Q}(\Lambda_{s}{\phi^{\prime }}_{s}|N_{s})\;d\xi_{s}+\frac{1}{2}\int_{\tau_{i-1}}^{\tau }E_{Q}\left(\sum_{j,k=1}^{n}\sum_{l=1}^{d}{\left(\frac{\partial^{2}\phi }{\partial x^{j}\partial x^{k}}\right)}_{s}g^{jl}g^{kl}|N_{s}\right)ds. \end{array}$$

Noting that $\int_{\tau_{i-1}}^{\tau }E_{Q}((\Lambda_{s}{\phi^{\prime }}_{s})|N_{s})\;d\xi_{s}=0$ and for notational convenience denoting the unnormalized conditional expectation operator, $E_{Q}({\left(\cdot \right)}_{\tau }\Lambda_{\tau }|N_{s})$ as $\Theta_{\tau }(\cdot )$ we arrive at the following equation:

A 7 $$\Theta_{\tau }(\phi )=\Theta_{i-1}(\phi )+\int_{\tau_{i-1}}^{\tau }\Theta_{s}(\phi h)\;d{\overset{⌣}{f}}_{s}+\frac{1}{2}\int_{\tau_{i-1}}^{\tau }\Theta_{s}\left(\sum_{j,k=1}^{n}\sum_{l=1}^{d}{\left(\frac{\partial^{2}\phi }{\partial x^{j}\partial x^{k}}\right)}_{s}g^{jl}g^{kl}\right)ds.$$

An incremental representation of equation (A 7) may be given as

A 8 $$d\Theta_{\tau }(\phi )=\Theta_{\tau }(\phi h)\;d{\overset{⌣}{f}}_{\tau }+\frac{1}{2}\Theta_{\tau }\left(\sum_{j,k=1}^{n}\sum_{l=1}^{d}\left(\frac{\partial^{2}\phi }{\partial x^{j}\partial x^{k}}\right)g^{jl}g^{kl}\right)d\tau .$$

In order to obtain the normalized conditional law, i.e. *π*_*τ*_(*ϕ*)=*Θ*_*τ*_(*ϕ*)/*Θ*_*τ*_(1), it is expanded using Ito's formula as given below:

A 9 $$d\pi_{\tau }(\phi )=\frac{d\Theta_{\tau }(\phi )}{\Theta_{\tau }(1)}+\Theta_{\tau }(\phi )\;d\left(\frac{1}{\Theta_{\tau }(1)}\right)+\left\langle d\Theta_{\tau }(\phi ),\;d\left(\frac{1}{\Theta_{\tau }(1)}\right)\right\rangle ,$$

where *d*(1/*Θ*_*τ*_(1)) may be expanded as

A 10 $$d\left(\frac{1}{\Theta_{\tau }(1)}\right)=-\frac{1}{\Theta_{\tau }^{2}(1)}\;d\Theta_{\tau }(1)+\frac{1}{\Theta_{\tau }^{3}(1)}\langle d\Theta_{\tau }(1),\;d\Theta_{\tau }(1)\rangle .$$

Putting *ϕ*=1 in equation (A 8), we get an Ito expansion for *dΘ*_*τ*_(1), which is given below:

A 11 $$d\Theta_{\tau }(1)=\Theta_{\tau }(h)\;d{\overset{⌣}{f}}_{\tau }.$$

Using equation (A 11) in equation (A 10),

A 12 $$d\left(\frac{1}{\Theta_{\tau }(1)}\right)=-\frac{\pi_{\tau }(h)}{\Theta_{\tau }(1)}\;d{\tilde{f}}_{\tau }+\frac{\pi_{\tau }^{2}(h)}{\Theta_{\tau }(1)}\;d\tau .$$

Using equation (A 8) and (A 12) in equation (A 9), we get

A 13 $$\begin{array}{rl} d\pi_{\tau }(\phi ) & =\pi_{\tau }(\phi h)\;d{\overset{⌣}{f}}_{\tau }+\frac{1}{2}\pi_{\tau }\left(\sum_{j,k=1}^{n}\sum_{l=1}^{d}\left(\frac{\partial^{2}\phi }{\partial x^{j}\partial x^{k}}\right)g^{jl}g^{kl}\right)d\tau \\ & \;+(-\pi_{\tau }(\phi )\pi_{\tau }(h)\;d{\overset{⌣}{f}}_{\tau }+\pi_{\tau }(\phi )\pi_{\tau }^{2}(h)\;d\tau )-\pi_{\tau }(\phi h)\pi_{\tau }(h)\;d\tau . \end{array}$$

Thus, we arrive at the extremal equation for the evolution of the normalized conditional estimate *π*_*τ*_(*ϕ*) given as

A 14 $$d\pi_{\tau }(\phi )=\frac{1}{2}\pi_{\tau }\left(\sum_{j,k=1}^{n}\sum_{l=1}^{d}\left(\frac{\partial^{2}\phi }{\partial x^{j}\partial x^{k}}\right)g^{jl}g^{kl}\right)d\tau +(\pi_{\tau }(\phi h)-\pi_{\tau }(\phi )\pi_{\tau }(h))(d{\overset{⌣}{f}}_{\tau }-\pi_{\tau }(h)\;d\tau ).$$

Remark A.2 Equation (A 7) finds its equivalence with the Zakai equation well known in stochastic filtering.

Remark A.3 Equation (A 14) has its parallel in the Kushner–Stratonovich equation, which is again well known in stochastic filtering.

Theorem A.4 *When ϕ(x)=x equation (*A 14*) may be rewritten as
*

$$d\pi_{\tau }(\text{x})=(\pi_{\tau }(\text{x}f)-\pi_{\tau }(\text{x})\pi_{\tau }(f)){\left(\rho_{\tau }\rho_{\tau }^{T}\right)}^{-1}(d{\overset{⌣}{f}}_{\tau }-\pi_{\tau }(f)\;d\tau ).$$

Proof. Since we are typically interested in the evolution of the conditional estimate of **x**_*τ*_, i.e. *ϕ* is the identity function, equation (A 14) may be simplified as

A 15 $$d\pi_{\tau }(\text{x})=(\pi_{\tau }(\text{x}h)-\pi_{\tau }(\text{x})\pi_{\tau }(h))(d{\overset{⌣}{f}}_{\tau }-\pi_{\tau }(h)\;d\tau ).$$

Replacing *h*(**x**_*τ*_) by $\rho_{\tau }^{-1}f({\text{x}}_{\tau })$ in equation (A 15), we get

A 16 $$d\pi_{\tau }(\text{x})=(\pi_{\tau }(\text{x}f)-\pi_{\tau }(\text{x})\pi_{\tau }(f)){\left(\rho_{\tau }\rho_{\tau }^{T}\right)}^{-1}(d{\overset{⌣}{f}}_{\tau }-\pi_{\tau }(f)\;d\tau ).$$

 ▪
