# Supplementary material for: A global optimization paradigm based on change of measures
Source: R Soc Open Sci. 2015 Jul 1;2(7):150123. doi: 10.1098/rsos.150123 (PMC4632581; doi:10.1098/rsos.150123)
Supplement: The supplementary material (Supplementay_material.tex) contains a detailed comparison, in tabular forms, of the proposed algorithm with CMA-ES. [file rsos150123supp1.pdf]

Supplementary Material for 'A global optimization paradigm based on change of measures'

Saikat Sarkar[1], Debasish Roy[1]* and Ram Mohan Vasu[2]

[1] *Computational Mechanics Lab, Department of Civil Engineering,*
[2] *Department of Instrumentation and Applied Physics Indian Institute of Science, Bangalore 560012, India*
**Corresponding author; email: royd@civil.iisc.ernet.in*

1. Introduction

A comparative performance of the CMA-ES and COMBEO in the context of the benchmark problems as considered in [1] for problem dimensions n=2,3,5,10 and 40 is reported in the following tables. Note that the tables corresponding to the problem dimension n=20 are already given in the main manuscript.

[1] *http://coco.gforge.inria.fr/doku.php.*

Preprint submitted to . *July 1, 2015*

Table I: n=2

F(I) index	Method	*EF(I)E*	error 1	error 2	error 3	error 4
$F(I)1$	*pseudo-code 2*$(CR = 0.1)$	3273	-8.94e-10	-8.94e-10	-8.94e-10	-8.94e-10
$F(I)1$	*pseudo-code 2*$(CR = rand)$	2455	5.42e-09	5.42e-09	5.42e-09	5.42e-09
$F(I)1$	*pseudo-code 2*$(CR = 0.9)$	2909	-7.87e-09	-7.87e-09	-7.87e-09	-7.87e-09
$F(I)1$	$CMA-ES$	504	-1.00e-08	-1.00e-08	-1.00e-08	-1.00e-08
$F(I)2$	*pseudo-code 2*$(CR = 0.1)$	4182	-6.14e-09	-6.14e-09	-6.14e-09	-6.14e-09
$F(I)2$	*pseudo-code 2*$(CR = rand)$	3818	4.82e-09	4.82e-09	4.82e-09	4.82e-09
$F(I)2$	*pseudo-code 2*$(CR = 0.9)$	3727	-7.46e-09	-7.46e-09	-7.46e-09	-7.46e-09
$F(I)2$	$CMA-ES$	901	-1.00e-08	-1.00e-08	-1.00e-08	-1.00e-08
$F(I)3$	*pseudo-code 2*$(CR = 0.1)$	4909	9.85e-09	9.85e-09	9.85e-09	9.85e-09
$F(I)3$	*pseudo-code 2*$(CR = rand)$	3818	7.75e-09	7.75e-09	7.75e-09	7.75e-09
$F(I)3$	*pseudo-code 2*$(CR = 0.9)$	4364	8.85e-09	8.85e-09	8.85e-09	8.85e-09
$F(I)3$	$CMA-ES$	729	-1.00e-08	-1.00e-08	-1.00e-08	-1.00e-08
$F(I)4$	*pseudo-code 2*$(CR = 0.1)$	6455	-9.05e-09	-9.05e-09	-9.05e-09	-9.05e-09
$F(I)4$	*pseudo-code 2*$(CR = rand)$	4545	-6.40e-09	-6.40e-09	-6.40e-09	-6.40e-09
$F(I)4$	*pseudo-code 2*$(CR = 0.9)$	4909	-2.09e-09	-2.09e-09	-2.09e-09	-2.09e-09
$F(I)4$	$CMA-ES$	714	1.90e+00	1.15e+00	3.22e+00	1.15e+00
$F(I)5$	*pseudo-code 2*$(CR = 0.1)$	1000	-1.00e-08	-1.00e-08	-1.00e-08	-1.00e-08
$F(I)5$	*pseudo-code 2*$(CR = rand)$	1091	-1.00e-08	-1.00e-08	-1.00e-08	-1.00e-08
$F(I)5$	*pseudo-code 2*$(CR = 0.9)$	1091	-1.00e-08	-1.00e-08	-1.00e-08	-1.00e-08
$F(I)5$	$CMA-ES$	58	-1.00e-08	-1.00e-08	-1.00e-08	-1.00e-08
$F(I)6$	*pseudo-code 2*$(CR = 0.1)$	1000000	1.12e-03	2.05e-06	9.31e-03	1.35e-04
$F(I)6$	*pseudo-code 2*$(CR = rand)$	192727	1.20e-05	7.01e-09	6.60e-05	7.01e-09
$F(I)6$	*pseudo-code 2*$(CR = 0.9)$	7364	1.46e-09	1.46e-09	1.46e-09	1.46e-09
$F(I)6$	$CMA-ES$	1019	-1.00e-08	-1.00e-08	-1.00e-08	-1.00e-08
$F(I)7$	*pseudo-code 2*$(CR = 0.1)$	5273	6.55e-09	6.55e-09	6.55e-09	6.55e-09
$F(I)7$	*pseudo-code 2*$(CR = rand)$	3364	1.89e-09	1.89e-09	1.89e-09	1.89e-09
$F(I)7$	*pseudo-code 2*$(CR = 0.9)$	2364	-8.78e-09	-8.78e-09	-8.78e-09	-8.78e-09
$F(I)7$	$CMA-ES$	322	9.04e-02	-1.00e-08	3.32e-01	-1.00e-08
$F(I)8$	*pseudo-code 2*$(CR = 0.1)$	27273	-9.47e-09	-9.47e-09	-9.47e-09	-9.47e-09
$F(I)8$	*pseudo-code 2*$(CR = rand)$	10909	-8.63e-10	-8.63e-10	-8.63e-10	-8.63e-10
$F(I)8$	*pseudo-code 2*$(CR = 0.9)$	7273	7.03e-09	7.03e-09	7.03e-09	7.03e-09
$F(I)8$	$CMA-ES$	933	-1.00e-08	-1.00e-08	-1.00e-08	-1.00e-08
$F(I)9$	*pseudo-code 2*$(CR = 0.1)$	14091	-2.81e-09	-2.81e-09	-2.81e-09	-2.81e-09
$F(I)9$	*pseudo-code 2*$(CR = rand)$	6909	-2.98e-09	-2.98e-09	-2.98e-09	-2.98e-09
$F(I)9$	*pseudo-code 2*$(CR = 0.9)$	6000	9.07e-09	9.07e-09	9.07e-09	9.07e-09
$F(I)9$	$CMA-ES$	943	-1.00e-08	-1.00e-08	-1.00e-08	-1.00e-08
$F(I)10$	*pseudo-code 2*$(CR = 0.1)$	1000000	1.97e-07	1.07e-08	7.64e-07	1.07e-08
$F(I)10$	*pseudo-code 2*$(CR = rand)$	49364	4.59e-09	4.59e-09	4.59e-09	4.59e-09
$F(I)10$	*pseudo-code 2*$(CR = 0.9)$	27091	-8.28e-09	-8.28e-09	-8.28e-09	-8.28e-09
$F(I)10$	$CMA-ES$	877	-1.00e-08	-1.00e-08	-1.00e-08	-1.00e-08
$F(I)11$	*pseudo-code 2*$(CR = 0.1)$	1000000	3.14e-06	6.64e-07	1.11e-05	9.19e-07
$F(I)11$	*pseudo-code 2*$(CR = rand)$	44727	9.65e-09	9.65e-09	9.65e-09	9.65e-09
$F(I)11$	*pseudo-code 2*$(CR = 0.9)$	31000	9.07e-09	9.07e-09	9.07e-09	9.07e-09
$F(I)11$	$CMA-ES$	913	-1.00e-08	-1.00e-08	-1.00e-08	-1.00e-08
$F(I)12$	*pseudo-code 2*$(CR = 0.1)$	11364	-6.48e-09	-6.48e-09	-6.48e-09	-6.48e-09
$F(I)12$	*pseudo-code 2*$(CR = rand)$	5727	1.70e-09	1.70e-09	1.70e-09	1.70e-09
$F(I)12$	*pseudo-code 2*$(CR = 0.9)$	5545	-8.34e-09	-8.34e-09	-8.34e-09	-8.34e-09
$F(I)12$	$CMA-ES$	921	-1.00e-08	-1.00e-08	-1.00e-08	-1.00e-08
$F(I)13$	*pseudo-code 2*$(CR = 0.1)$	1000000	1.06e-05	1.63e-06	8.16e-05	4.02e-06
$F(I)13$	*pseudo-code 2*$(CR = rand)$	1000000	3.52e-07	1.43e-07	8.75e-07	2.82e-07
$F(I)13$	*pseudo-code 2*$(CR = 0.9)$	1000000	1.34e-07	6.40e-08	3.82e-07	6.40e-08
$F(I)13$	$CMA-ES$	1253	-1.00e-08	-1.00e-08	-9.99e-09	-1.00e-08

Table II: n=2

F(I) index	Method	*EF(I)E*	error 1	error 2	error 3	error 4
$F(I)14$	*pseudo-code 2*$(CR = 0.1)$	140455	6.71e-09	4.87e-09	2.51e-08	4.87e-09
$F(I)14$	*pseudo-code 2*$(CR = rand)$	24818	-7.23e-09	-7.23e-09	-7.23e-09	-7.23e-09
$F(I)14$	*pseudo-code 2*$(CR = 0.9)$	16545	-7.30e-09	-7.30e-09	-7.30e-09	-7.30e-09
$F(I)14$	$CMA - ES$	1164	-1.00e-08	-1.00e-08	-1.00e-08	-1.00e-08
$F(I)15$	*pseudo-code 2*$(CR = 0.1)$	10091	8.21e-09	8.21e-09	8.21e-09	8.21e-09
$F(I)15$	*pseudo-code 2*$(CR = rand)$	7091	-1.98e-10	-1.98e-10	-1.98e-10	-1.98e-10
$F(I)15$	*pseudo-code 2*$(CR = 0.9)$	5909	-3.64e-09	-3.64e-09	-3.64e-09	-3.64e-09
$F(I)15$	$CMA - ES$	691	1.87e+00	-1.00e-08	1.59e+01	-1.00e-08
$F(I)16$	*pseudo-code 2*$(CR = 0.1)$	44273	6.61e-09	6.61e-09	6.61e-09	6.61e-09
$F(I)16$	*pseudo-code 2*$(CR = rand)$	17000	-9.95e-09	-9.95e-09	-9.95e-09	-9.95e-09
$F(I)16$	*pseudo-code 2*$(CR = 0.9)$	26182	6.75e-09	6.75e-09	6.75e-09	6.75e-09
$F(I)16$	$CMA - ES$	694	3.90e-05	-1.00e-08	4.30e-04	-1.00e-08
$F(I)17$	*pseudo-code 2*$(CR = 0.1)$	11455	3.20e-09	3.20e-09	3.20e-09	3.20e-09
$F(I)17$	*pseudo-code 2*$(CR = rand)$	8000	-1.62e-09	-1.62e-09	-1.62e-09	-1.62e-09
$F(I)17$	*pseudo-code 2*$(CR = 0.9)$	7182	8.16e-09	8.16e-09	8.16e-09	8.16e-09
$F(I)17$	$CMA - ES$	789	5.53e-04	2.40e-05	3.88e-03	2.40e-05
$F(I)18$	*pseudo-code 2*$(CR = 0.1)$	32364	4.34e-09	4.34e-09	4.34e-09	4.34e-09
$F(I)18$	*pseudo-code 2*$(CR = rand)$	18000	5.70e-09	5.70e-09	5.70e-09	5.70e-09
$F(I)18$	*pseudo-code 2*$(CR = 0.9)$	12091	7.53e-09	7.53e-09	7.53e-09	7.53e-09
$F(I)18$	$CMA - ES$	783	2.85e-01	1.11e-05	3.13e+00	1.11e-05
$F(I)19$	*pseudo-code 2*$(CR = 0.1)$	7909	-3.73e-09	-3.73e-09	-3.73e-09	-3.73e-09
$F(I)19$	*pseudo-code 2*$(CR = rand)$	6455	-9.22e-09	-9.22e-09	-9.22e-09	-9.22e-09
$F(I)19$	*pseudo-code 2*$(CR = 0.9)$	5455	-7.69e-09	-7.69e-09	-7.69e-09	-7.69e-09
$F(I)19$	$CMA - ES$	1001	2.32e-02	-1.00e-08	2.55e-01	-1.00e-08
$F(I)20$	*pseudo-code 2*$(CR = 0.1)$	6636	-6.19e-09	-6.19e-09	-6.19e-09	-6.19e-09
$F(I)20$	*pseudo-code 2*$(CR = rand)$	4545	3.89e-09	3.89e-09	3.89e-09	3.89e-09
$F(I)20$	*pseudo-code 2*$(CR = 0.9)$	4455	-6.91e-09	-6.91e-09	-6.91e-09	-6.91e-09
$F(I)20$	$CMA - ES$	687	3.25e-01	-1.00e-08	1.25e+00	-1.00e-08
$F(I)21$	*pseudo-code 2*$(CR = 0.1)$	4636	-4.30e-09	-4.30e-09	-4.30e-09	-4.30e-09
$F(I)21$	*pseudo-code 2*$(CR = rand)$	3273	1.29e-09	1.29e-09	1.29e-09	1.29e-09
$F(I)21$	*pseudo-code 2*$(CR = 0.9)$	2364	-6.82e-09	-6.82e-09	-6.82e-09	-6.82e-09
$F(I)21$	$CMA - ES$	484	-1.00e-08	-1.00e-08	-1.00e-08	-1.00e-08
$F(I)22$	*pseudo-code 2*$(CR = 0.1)$	8818	-7.98e-09	-7.98e-09	-7.98e-09	-7.98e-09
$F(I)22$	*pseudo-code 2*$(CR = rand)$	4273	2.29e-09	2.29e-09	2.29e-09	2.29e-09
$F(I)22$	*pseudo-code 2*$(CR = 0.9)$	3364	-7.96e-09	-7.96e-09	-7.96e-09	-7.96e-09
$F(I)22$	$CMA - ES$	575	1.80e-01	-1.00e-08	1.95e+00	-1.00e-08
$F(I)23$	*pseudo-code 2*$(CR = 0.1)$	1000000	1.61e-02	1.11e-02	6.63e-02	1.11e-02
$F(I)23$	*pseudo-code 2*$(CR = rand)$	1000000	4.57e-02	2.82e-02	9.97e-02	3.04e-02
$F(I)23$	*pseudo-code 2*$(CR = 0.9)$	1000000	2.54e-02	1.27e-02	4.06e-02	1.27e-02
$F(I)23$	$CMA - ES$	1126	4.95e-01	-8.93e-09	6.81e-01	6.81e-01
$F(I)24$	*pseudo-code 2*$(CR = 0.1)$	1000000	7.89e-03	1.69e-04	3.48e-02	2.15e-03
$F(I)24$	*pseudo-code 2*$(CR = rand)$	48000	3.89e-09	3.89e-09	3.89e-09	3.89e-09
$F(I)24$	*pseudo-code 2*$(CR = 0.9)$	30364	-5.11e-09	-5.11e-09	-5.11e-09	-5.11e-09
$F(I)24$	$CMA - ES$	1059	6.35e-01	9.29e-02	3.14e+00	9.29e-02

Table III: n=3

F(I) index	Method	*EF(I)E*	error 1	error 2	error 3	error 4
$F(I)1$	*pseudo-code 2*$(CR = 0.1)$	6000	-9.68e-09	-9.68e-09	-9.68e-09	-9.68e-09
$F(I)1$	*pseudo-code 2*$(CR = rand)$	4545	9.30e-09	9.30e-09	9.30e-09	9.30e-09
$F(I)1$	*pseudo-code 2*$(CR = 0.9)$	4273	-6.05e-09	-6.05e-09	-6.05e-09	-6.05e-09
$F(I)1$	$CMA - ES$	784	-1.00e-08	-1.00e-08	-1.00e-08	-1.00e-08
$F(I)2$	*pseudo-code 2*$(CR = 0.1)$	7455	9.75e-10	9.75e-10	9.75e-10	9.75e-10
$F(I)2$	*pseudo-code 2*$(CR = rand)$	5727	-4.00e-09	-4.00e-09	-4.00e-09	-4.00e-09
$F(I)2$	*pseudo-code 2*$(CR = 0.9)$	5909	-3.08e-09	-3.08e-09	-3.08e-09	-3.08e-09
$F(I)2$	$CMA - ES$	1487	-1.00e-08	-1.00e-08	-1.00e-08	-1.00e-08
$F(I)3$	*pseudo-code 2*$(CR = 0.1)$	8273	3.69e-09	3.69e-09	3.69e-09	3.69e-09
$F(I)3$	*pseudo-code 2*$(CR = rand)$	7273	4.13e-09	4.13e-09	4.13e-09	4.13e-09
$F(I)3$	*pseudo-code 2*$(CR = 0.9)$	8091	4.67e-11	4.66e-11	4.66e-11	4.66e-11
$F(I)3$	$CMA - ES$	1056	1.86e+00	9.95e-01	5.51e+00	1.99e+00
$F(I)4$	*pseudo-code 2*$(CR = 0.1)$	8818	-7.92e-09	-7.92e-09	-7.92e-09	-7.92e-09
$F(I)4$	*pseudo-code 2*$(CR = rand)$	8545	-7.19e-09	-7.19e-09	-7.19e-09	-7.19e-09
$F(I)4$	*pseudo-code 2*$(CR = 0.9)$	10636	1.25e-09	1.25e-09	1.25e-09	1.25e-09
$F(I)4$	$CMA - ES$	1172	1.63e+00	-1.00e-08	3.98e+00	1.99e+00
$F(I)5$	*pseudo-code 2*$(CR = 0.1)$	1545	-1.00e-08	-1.00e-08	-1.00e-08	-1.00e-08
$F(I)5$	*pseudo-code 2*$(CR = rand)$	1455	-1.00e-08	-1.00e-08	-1.00e-08	-1.00e-08
$F(I)5$	*pseudo-code 2*$(CR = 0.9)$	1364	-1.00e-08	-1.00e-08	-1.00e-08	-1.00e-08
$F(I)5$	$CMA - ES$	55	-1.00e-08	-1.00e-08	-1.00e-08	-1.00e-08
$F(I)6$	*pseudo-code 2*$(CR = 0.1)$	133455	9.15e-08	4.62e-09	9.60e-07	4.62e-09
$F(I)6$	*pseudo-code 2*$(CR = rand)$	8182	2.25e-09	2.25e-09	2.25e-09	2.25e-09
$F(I)6$	*pseudo-code 2*$(CR = 0.9)$	8273	6.30e-09	6.30e-09	6.30e-09	6.30e-09
$F(I)6$	$CMA - ES$	1496	-1.00e-08	-1.00e-08	-1.00e-08	-1.00e-08
$F(I)7$	*pseudo-code 2*$(CR = 0.1)$	6727	2.42e-09	2.42e-09	2.42e-09	2.42e-09
$F(I)7$	*pseudo-code 2*$(CR = rand)$	4273	-2.23e-09	-2.23e-09	-2.23e-09	-2.23e-09
$F(I)7$	*pseudo-code 2*$(CR = 0.9)$	3000	-4.53e-09	-4.53e-09	-4.53e-09	-4.53e-09
$F(I)7$	$CMA - ES$	631	3.50e-02	-1.00e-08	1.28e-01	-1.00e-08
$F(I)8$	*pseudo-code 2*$(CR = 0.1)$	1000000	9.53e-06	9.53e-06	9.53e-06	9.53e-06
$F(I)8$	*pseudo-code 2*$(CR = rand)$	39000	1.43e-09	1.43e-09	1.43e-09	1.43e-09
$F(I)8$	*pseudo-code 2*$(CR = 0.9)$	17818	1.41e-09	1.41e-09	1.41e-09	1.41e-09
$F(I)8$	$CMA - ES$	1440	-1.00e-08	-1.00e-08	-1.00e-08	-1.00e-08
$F(I)9$	*pseudo-code 2*$(CR = 0.1)$	164818	2.28e-05	4.83e-09	2.51e-04	4.83e-09
$F(I)9$	*pseudo-code 2*$(CR = rand)$	32273	7.45e-09	7.45e-09	7.45e-09	7.45e-09
$F(I)9$	*pseudo-code 2*$(CR = 0.9)$	16455	1.28e-09	1.28e-09	1.28e-09	1.28e-09
$F(I)9$	$CMA - ES$	1687	-1.00e-08	-1.00e-08	-1.00e-08	-1.00e-08
$F(I)10$	*pseudo-code 2*$(CR = 0.1)$	1000000	5.96e-03	5.96e-03	5.96e-03	5.96e-03
$F(I)10$	*pseudo-code 2*$(CR = rand)$	1000000	3.70e-07	9.41e-08	1.04e-06	3.54e-07
$F(I)10$	*pseudo-code 2*$(CR = 0.9)$	70727	5.16e-09	5.16e-09	5.16e-09	5.16e-09
$F(I)10$	$CMA - ES$	1451	-1.00e-08	-1.00e-08	-1.00e-08	-1.00e-08
$F(I)11$	*pseudo-code 2*$(CR = 0.1)$	1000000	3.28e-02	1.79e-02	6.89e-02	1.79e-02
$F(I)11$	*pseudo-code 2*$(CR = rand)$	1000000	1.10e-03	2.22e-04	4.49e-03	2.22e-04
$F(I)11$	*pseudo-code 2*$(CR = 0.9)$	1000000	2.67e-05	1.20e-05	3.25e-05	3.19e-05
$F(I)11$	$CMA - ES$	1535	-1.00e-08	-1.00e-08	-1.00e-08	-1.00e-08
$F(I)12$	*pseudo-code 2*$(CR = 0.1)$	1000000	1.05e+00	1.01e-01	2.05e+00	1.01e+00
$F(I)12$	*pseudo-code 2*$(CR = rand)$	1000000	1.20e-03	8.40e-04	1.40e-03	1.40e-03
$F(I)12$	*pseudo-code 2*$(CR = 0.9)$	1000000	2.76e-05	2.62e-05	4.14e-05	2.62e-05
$F(I)12$	$CMA - ES$	4404	-1.00e-08	-1.00e-08	-1.00e-08	-1.00e-08

Table IV: n=3

F(I) index	Method	*EF(I)E*	error 1	error 2	error 3	error 4
$F(I)13$	*pseudo-code 2*$(CR = 0.1)$	1000000	5.23e-04	5.23e-04	5.23e-04	5.23e-04
$F(I)13$	*pseudo-code 2*$(CR = rand)$	1000000	3.91e-04	1.50e-04	6.81e-04	3.25e-04
$F(I)13$	*pseudo-code 2*$(CR = 0.9)$	1000000	1.18e-04	8.17e-05	1.98e-04	1.06e-04
$F(I)13$	$CMA - ES$	2246	-1.00e-08	-1.00e-08	-1.00e-08	-1.00e-08
$F(I)14$	*pseudo-code 2*$(CR = 0.1)$	1000000	6.19e-07	4.37e-07	2.32e-06	4.37e-07
$F(I)14$	*pseudo-code 2*$(CR = rand)$	715364	2.36e-08	8.54e-09	3.22e-08	3.22e-08
$F(I)14$	*pseudo-code 2*$(CR = 0.9)$	63455	1.53e-10	1.53e-10	1.53e-10	1.53e-10
$F(I)14$	$CMA - ES$	2063	-1.00e-08	-1.00e-08	-1.00e-08	-1.00e-08
$F(I)15$	*pseudo-code 2*$(CR = 0.1)$	42364	9.18e-09	9.18e-09	9.18e-09	9.18e-09
$F(I)15$	*pseudo-code 2*$(CR = rand)$	15727	-4.07e-09	-4.07e-09	-4.07e-09	-4.07e-09
$F(I)15$	*pseudo-code 2*$(CR = 0.9)$	12455	-3.70e-09	-3.70e-09	-3.70e-09	-3.70e-09
$F(I)15$	$CMA - ES$	1127	1.32e+00	9.95e-01	1.99e+00	9.95e-01
$F(I)16$	*pseudo-code 2*$(CR = 0.1)$	166545	3.89e-04	7.29e-09	4.28e-03	7.29e-09
$F(I)16$	*pseudo-code 2*$(CR = rand)$	26091	-2.25e-09	-2.25e-09	-2.25e-09	-2.25e-09
$F(I)16$	*pseudo-code 2*$(CR = 0.9)$	34364	-4.92e-09	-4.92e-09	-4.92e-09	-4.92e-09
$F(I)16$	$CMA - ES$	1393	2.20e-02	-1.00e-08	4.03e-02	4.03e-02
$F(I)17$	*pseudo-code 2*$(CR = 0.1)$	33000	9.18e-09	9.18e-09	9.18e-09	9.18e-09
$F(I)17$	*pseudo-code 2*$(CR = rand)$	15364	3.92e-09	3.92e-09	3.92e-09	3.92e-09
$F(I)17$	*pseudo-code 2*$(CR = 0.9)$	13091	-7.70e-10	-7.70e-10	-7.70e-10	-7.70e-10
$F(I)17$	$CMA - ES$	1407	1.28e-05	-9.99e-09	1.05e-04	7.19e-06
$F(I)18$	*pseudo-code 2*$(CR = 0.1)$	1000000	2.21e-05	4.14e-06	1.93e-04	5.28e-06
$F(I)18$	*pseudo-code 2*$(CR = rand)$	40636	5.95e-09	5.95e-09	5.95e-09	5.95e-09
$F(I)18$	*pseudo-code 2*$(CR = 0.9)$	27273	-5.34e-09	-5.34e-09	-5.34e-09	-5.34e-09
$F(I)18$	$CMA - ES$	1501	7.97e-01	7.19e-06	8.00e+00	1.86e-03
$F(I)19$	*pseudo-code 2*$(CR = 0.1)$	1000000	1.58e-05	3.19e-06	1.42e-04	3.19e-06
$F(I)19$	*pseudo-code 2*$(CR = rand)$	58000	3.70e-09	3.70e-09	3.70e-09	3.70e-09
$F(I)19$	*pseudo-code 2*$(CR = 0.9)$	35455	6.65e-09	6.65e-09	6.65e-09	6.65e-09
$F(I)19$	$CMA - ES$	6287	1.56e-01	9.42e-02	3.61e-01	9.42e-02
$F(I)20$	*pseudo-code 2*$(CR = 0.1)$	15636	-5.18e-09	-5.18e-09	-5.18e-09	-5.18e-09
$F(I)20$	*pseudo-code 2*$(CR = rand)$	7818	6.59e-11	6.58e-11	6.58e-11	6.58e-11
$F(I)20$	*pseudo-code 2*$(CR = 0.9)$	7727	-7.06e-09	-7.06e-09	-7.06e-09	-7.06e-09
$F(I)20$	$CMA - ES$	1026	8.89e-01	7.24e-01	1.63e+00	7.24e-01
$F(I)21$	*pseudo-code 2*$(CR = 0.1)$	9364	2.55e-09	2.55e-09	2.55e-09	2.55e-09
$F(I)21$	*pseudo-code 2*$(CR = rand)$	5273	6.51e-09	6.51e-09	6.51e-09	6.51e-09
$F(I)21$	*pseudo-code 2*$(CR = 0.9)$	4273	-2.99e-09	-2.99e-09	-2.99e-09	-2.99e-09
$F(I)21$	$CMA - ES$	806	8.45e-02	-1.00e-08	9.30e-01	-1.00e-08
$F(I)22$	*pseudo-code 2*$(CR = 0.1)$	1000000	2.01e-04	1.34e-04	7.74e-04	1.34e-04
$F(I)22$	*pseudo-code 2*$(CR = rand)$	10455	2.96e-09	2.96e-09	2.96e-09	2.96e-09
$F(I)22$	*pseudo-code 2*$(CR = 0.9)$	7818	3.09e-09	3.09e-09	3.09e-09	3.09e-09
$F(I)22$	$CMA - ES$	764	8.74e-04	-1.00e-08	9.62e-03	-1.00e-08
$F(I)23$	*pseudo-code 2*$(CR = 0.1)$	1000000	1.32e-01	1.22e-01	2.35e-01	1.22e-01
$F(I)23$	*pseudo-code 2*$(CR = rand)$	1000000	1.74e-01	1.62e-01	2.07e-01	1.62e-01
$F(I)23$	*pseudo-code 2*$(CR = 0.9)$	1000000	1.27e-01	8.05e-02	2.20e-01	1.18e-01
$F(I)23$	$CMA - ES$	1254	2.30e-01	1.10e-01	1.43e+00	1.10e-01
$F(I)24$	*pseudo-code 2*$(CR = 0.1)$	1000000	2.10e+00	4.27e-01	3.09e+00	2.38e+00
$F(I)24$	*pseudo-code 2*$(CR = rand)$	1000000	7.95e-01	5.67e-01	8.81e-01	8.81e-01
$F(I)24$	*pseudo-code 2*$(CR = 0.9)$	1000000	3.70e-01	2.21e-01	7.21e-01	2.83e-01
$F(I)24$	$CMA - ES$	1531	1.06e+00	6.26e-01	3.27e+00	8.96e-01

Table V: n=5

F(I) index	Method	*EF(I)E*	error 1	error 2	error 3	error 4
$F(I)1$	*pseudo-code 2*$(CR = 0.1)$	6000	-9.68e-09	-9.68e-09	-9.68e-09	-9.68e-09
$F(I)1$	*pseudo-code 2*$(CR = rand)$	4545	9.30e-09	9.30e-09	9.30e-09	9.30e-09
$F(I)1$	*pseudo-code 2*$(CR = 0.9)$	4273	-6.05e-09	-6.05e-09	-6.05e-09	-6.05e-09
$F(I)1$	$CMA - ES$	784	-1.00e-08	-1.00e-08	-1.00e-08	-1.00e-08
$F(I)2$	*pseudo-code 2*$(CR = 0.1)$	7455	9.75e-10	9.75e-10	9.75e-10	9.75e-10
$F(I)2$	*pseudo-code 2*$(CR = rand)$	5727	-4.00e-09	-4.00e-09	-4.00e-09	-4.00e-09
$F(I)2$	*pseudo-code 2*$(CR = 0.9)$	5909	-3.08e-09	-3.08e-09	-3.08e-09	-3.08e-09
$F(I)2$	$CMA - ES$	1487	-1.00e-08	-1.00e-08	-1.00e-08	-1.00e-08
$F(I)3$	*pseudo-code 2*$(CR = 0.1)$	8273	3.69e-09	3.69e-09	3.69e-09	3.69e-09
$F(I)3$	*pseudo-code 2*$(CR = rand)$	7273	4.13e-09	4.13e-09	4.13e-09	4.13e-09
$F(I)3$	*pseudo-code 2*$(CR = 0.9)$	8091	4.67e-11	4.66e-11	4.66e-11	4.66e-11
$F(I)3$	$CMA - ES$	1056	1.86e+00	9.95e-01	5.51e+00	1.99e+00
$F(I)4$	*pseudo-code 2*$(CR = 0.1)$	8818	-7.92e-09	-7.92e-09	-7.92e-09	-7.92e-09
$F(I)4$	*pseudo-code 2*$(CR = rand)$	8545	-7.19e-09	-7.19e-09	-7.19e-09	-7.19e-09
$F(I)4$	*pseudo-code 2*$(CR = 0.9)$	10636	1.25e-09	1.25e-09	1.25e-09	1.25e-09
$F(I)4$	$CMA - ES$	1172	1.63e+00	-1.00e-08	3.98e+00	1.99e+00
$F(I)5$	*pseudo-code 2*$(CR = 0.1)$	1545	-1.00e-08	-1.00e-08	-1.00e-08	-1.00e-08
$F(I)5$	*pseudo-code 2*$(CR = rand)$	1455	-1.00e-08	-1.00e-08	-1.00e-08	-1.00e-08
$F(I)5$	*pseudo-code 2*$(CR = 0.9)$	1364	-1.00e-08	-1.00e-08	-1.00e-08	-1.00e-08
$F(I)5$	$CMA - ES$	55	-1.00e-08	-1.00e-08	-1.00e-08	-1.00e-08
$F(I)6$	*pseudo-code 2*$(CR = 0.1)$	133455	9.15e-08	4.62e-09	9.60e-07	4.62e-09
$F(I)6$	*pseudo-code 2*$(CR = rand)$	8182	2.25e-09	2.25e-09	2.25e-09	2.25e-09
$F(I)6$	*pseudo-code 2*$(CR = 0.9)$	8273	6.30e-09	6.30e-09	6.30e-09	6.30e-09
$F(I)6$	$CMA - ES$	1496	-1.00e-08	-1.00e-08	-1.00e-08	-1.00e-08
$F(I)7$	*pseudo-code 2*$(CR = 0.1)$	6727	2.42e-09	2.42e-09	2.42e-09	2.42e-09
$F(I)7$	*pseudo-code 2*$(CR = rand)$	4273	-2.23e-09	-2.23e-09	-2.23e-09	-2.23e-09
$F(I)7$	*pseudo-code 2*$(CR = 0.9)$	3000	-4.53e-09	-4.53e-09	-4.53e-09	-4.53e-09
$F(I)7$	$CMA - ES$	631	3.50e-02	-1.00e-08	1.28e-01	-1.00e-08
$F(I)8$	*pseudo-code 2*$(CR = 0.1)$	1000000	9.53e-06	9.53e-06	9.53e-06	9.53e-06
$F(I)8$	*pseudo-code 2*$(CR = rand)$	39000	1.43e-09	1.43e-09	1.43e-09	1.43e-09
$F(I)8$	*pseudo-code 2*$(CR = 0.9)$	17818	1.41e-09	1.41e-09	1.41e-09	1.41e-09
$F(I)8$	$CMA - ES$	1440	-1.00e-08	-1.00e-08	-1.00e-08	-1.00e-08
$F(I)9$	*pseudo-code 2*$(CR = 0.1)$	164818	2.28e-05	4.83e-09	2.51e-04	4.83e-09
$F(I)9$	*pseudo-code 2*$(CR = rand)$	32273	7.45e-09	7.45e-09	7.45e-09	7.45e-09
$F(I)9$	*pseudo-code 2*$(CR = 0.9)$	16455	1.28e-09	1.28e-09	1.28e-09	1.28e-09
$F(I)9$	$CMA - ES$	1687	-1.00e-08	-1.00e-08	-1.00e-08	-1.00e-08
$F(I)10$	*pseudo-code 2*$(CR = 0.1)$	1000000	5.96e-03	5.96e-03	5.96e-03	5.96e-03
$F(I)10$	*pseudo-code 2*$(CR = rand)$	1000000	3.70e-07	9.41e-08	1.04e-06	3.54e-07
$F(I)10$	*pseudo-code 2*$(CR = 0.9)$	70727	5.16e-09	5.16e-09	5.16e-09	5.16e-09
$F(I)10$	$CMA - ES$	1451	-1.00e-08	-1.00e-08	-1.00e-08	-1.00e-08
$F(I)11$	*pseudo-code 2*$(CR = 0.1)$	1000000	3.28e-02	1.79e-02	6.89e-02	1.79e-02
$F(I)11$	*pseudo-code 2*$(CR = rand)$	1000000	1.10e-03	2.22e-04	4.49e-03	2.22e-04
$F(I)11$	*pseudo-code 2*$(CR = 0.9)$	1000000	2.67e-05	1.20e-05	3.25e-05	3.19e-05
$F(I)11$	$CMA - ES$	1535	-1.00e-08	-1.00e-08	-1.00e-08	-1.00e-08
$F(I)12$	*pseudo-code 2*$(CR = 0.1)$	1000000	1.05e+00	1.01e-01	2.05e+00	1.01e+00
$F(I)12$	*pseudo-code 2*$(CR = rand)$	1000000	1.20e-03	8.40e-04	1.40e-03	1.40e-03
$F(I)12$	*pseudo-code 2*$(CR = 0.9)$	1000000	2.76e-05	2.62e-05	4.14e-05	2.62e-05
$F(I)12$	$CMA - ES$	4404	-1.00e-08	-1.00e-08	-1.00e-08	-1.00e-08

Table VI: Dimension 5

F(I) index	Method	*EF(I)E*	error 1	error 2	error 3	error 4
$F(I)13$	*pseudo-code 2*$(CR = 0.1)$	1000000	5.23e-04	5.23e-04	5.23e-04	5.23e-04
$F(I)13$	*pseudo-code 2*$(CR = rand)$	1000000	3.91e-04	1.50e-04	6.81e-04	3.25e-04
$F(I)13$	*pseudo-code 2*$(CR = 0.9)$	1000000	1.18e-04	8.17e-05	1.98e-04	1.06e-04
$F(I)13$	$CMA - ES$	2246	-1.00e-08	-1.00e-08	-1.00e-08	-1.00e-08
$F(I)14$	*pseudo-code 2*$(CR = 0.1)$	1000000	6.19e-07	4.37e-07	2.32e-06	4.37e-07
$F(I)14$	*pseudo-code 2*$(CR = rand)$	715364	2.36e-08	8.54e-09	3.22e-08	3.22e-08
$F(I)14$	*pseudo-code 2*$(CR = 0.9)$	63455	1.53e-10	1.53e-10	1.53e-10	1.53e-10
$F(I)14$	$CMA - ES$	2063	-1.00e-08	-1.00e-08	-1.00e-08	-1.00e-08
$F(I)15$	*pseudo-code 2*$(CR = 0.1)$	42364	9.18e-09	9.18e-09	9.18e-09	9.18e-09
$F(I)15$	*pseudo-code 2*$(CR = rand)$	15727	-4.07e-09	-4.07e-09	-4.07e-09	-4.07e-09
$F(I)15$	*pseudo-code 2*$(CR = 0.9)$	12455	-3.70e-09	-3.70e-09	-3.70e-09	-3.70e-09
$F(I)15$	$CMA - ES$	1127	1.32e+00	9.95e-01	1.99e+00	9.95e-01
$F(I)16$	*pseudo-code 2*$(CR = 0.1)$	166545	3.89e-04	7.29e-09	4.28e-03	7.29e-09
$F(I)16$	*pseudo-code 2*$(CR = rand)$	26091	-2.25e-09	-2.25e-09	-2.25e-09	-2.25e-09
$F(I)16$	*pseudo-code 2*$(CR = 0.9)$	34364	-4.92e-09	-4.92e-09	-4.92e-09	-4.92e-09
$F(I)16$	$CMA - ES$	1393	2.20e-02	-1.00e-08	4.03e-02	4.03e-02
$F(I)17$	*pseudo-code 2*$(CR = 0.1)$	33000	9.18e-09	9.18e-09	9.18e-09	9.18e-09
$F(I)17$	*pseudo-code 2*$(CR = rand)$	15364	3.92e-09	3.92e-09	3.92e-09	3.92e-09
$F(I)17$	*pseudo-code 2*$(CR = 0.9)$	13091	-7.70e-10	-7.70e-10	-7.70e-10	-7.70e-10
$F(I)17$	$CMA - ES$	1407	1.28e-05	-9.99e-09	1.05e-04	7.19e-06
$F(I)18$	*pseudo-code 2*$(CR = 0.1)$	1000000	2.21e-05	4.14e-06	1.93e-04	5.28e-06
$F(I)18$	*pseudo-code 2*$(CR = rand)$	40636	5.95e-09	5.95e-09	5.95e-09	5.95e-09
$F(I)18$	*pseudo-code 2*$(CR = 0.9)$	27273	-5.34e-09	-5.34e-09	-5.34e-09	-5.34e-09
$F(I)18$	$CMA - ES$	1501	7.97e-01	7.19e-06	8.00e+00	1.86e-03
$F(I)19$	*pseudo-code 2*$(CR = 0.1)$	1000000	1.58e-05	3.19e-06	1.42e-04	3.19e-06
$F(I)19$	*pseudo-code 2*$(CR = rand)$	58000	3.70e-09	3.70e-09	3.70e-09	3.70e-09
$F(I)19$	*pseudo-code 2*$(CR = 0.9)$	35455	6.65e-09	6.65e-09	6.65e-09	6.65e-09
$F(I)19$	$CMA - ES$	6287	1.56e-01	9.42e-02	3.61e-01	9.42e-02
$F(I)20$	*pseudo-code 2*$(CR = 0.1)$	15636	-5.18e-09	-5.18e-09	-5.18e-09	-5.18e-09
$F(I)20$	*pseudo-code 2*$(CR = rand)$	7818	6.59e-11	6.58e-11	6.58e-11	6.58e-11
$F(I)20$	*pseudo-code 2*$(CR = 0.9)$	7727	-7.06e-09	-7.06e-09	-7.06e-09	-7.06e-09
$F(I)20$	$CMA - ES$	1026	8.89e-01	7.24e-01	1.63e+00	7.24e-01
$F(I)21$	*pseudo-code 2*$(CR = 0.1)$	9364	2.55e-09	2.55e-09	2.55e-09	2.55e-09
$F(I)21$	*pseudo-code 2*$(CR = rand)$	5273	6.51e-09	6.51e-09	6.51e-09	6.51e-09
$F(I)21$	*pseudo-code 2*$(CR = 0.9)$	4273	-2.99e-09	-2.99e-09	-2.99e-09	-2.99e-09
$F(I)21$	$CMA - ES$	806	8.45e-02	-1.00e-08	9.30e-01	-1.00e-08
$F(I)22$	*pseudo-code 2*$(CR = 0.1)$	1000000	2.01e-04	1.34e-04	7.74e-04	1.34e-04
$F(I)22$	*pseudo-code 2*$(CR = rand)$	10455	2.96e-09	2.96e-09	2.96e-09	2.96e-09
$F(I)22$	*pseudo-code 2*$(CR = 0.9)$	7818	3.09e-09	3.09e-09	3.09e-09	3.09e-09
$F(I)22$	$CMA - ES$	764	8.74e-04	-1.00e-08	9.62e-03	-1.00e-08
$F(I)23$	*pseudo-code 2*$(CR = 0.1)$	1000000	1.32e-01	1.22e-01	2.35e-01	1.22e-01
$F(I)23$	*pseudo-code 2*$(CR = rand)$	1000000	1.74e-01	1.62e-01	2.07e-01	1.62e-01
$F(I)23$	*pseudo-code 2*$(CR = 0.9)$	1000000	1.27e-01	8.05e-02	2.20e-01	1.18e-01
$F(I)23$	$CMA - ES$	1254	2.30e-01	1.10e-01	1.43e+00	1.10e-01
$F(I)24$	*pseudo-code 2*$(CR = 0.1)$	1000000	2.10e+00	4.27e-01	3.09e+00	2.38e+00
$F(I)24$	*pseudo-code 2*$(CR = rand)$	1000000	7.95e-01	5.67e-01	8.81e-01	8.81e-01
$F(I)24$	*pseudo-code 2*$(CR = 0.9)$	1000000	3.70e-01	2.21e-01	7.21e-01	2.83e-01
$F(I)24$	$CMA - ES$	1531	1.06e+00	6.26e-01	3.27e+00	8.96e-01

Table VII: n= 10

F(I) index	Method	*EF(I)E*	error 1	error 2	error 3	error 4
$F(I)1$	*pseudo-code 2*$(CR = 0.1)$	16727	7.51e-09	7.51e-09	7.51e-09	7.51e-09
$F(I)1$	*pseudo-code 2*$(CR = rand)$	16000	8.34e-09	8.34e-09	8.34e-09	8.34e-09
$F(I)1$	*pseudo-code 2*$(CR = 0.9)$	18727	9.01e-09	9.01e-09	9.01e-09	9.01e-09
$F(I)1$	$CMA - ES$	2437	-1.00e-08	-1.00e-08	-1.00e-08	-1.00e-08
$F(I)2$	*pseudo-code 2*$(CR = 0.1)$	22818	8.86e-09	8.86e-09	8.86e-09	8.86e-09
$F(I)2$	*pseudo-code 2*$(CR = rand)$	21727	-8.94e-10	-8.94e-10	-8.94e-10	-8.94e-10
$F(I)2$	*pseudo-code 2*$(CR = 0.9)$	26545	4.32e-09	4.32e-09	4.32e-09	4.32e-09
$F(I)2$	$CMA - ES$	7000	-1.00e-08	-1.00e-08	-1.00e-08	-1.00e-08
$F(I)3$	*pseudo-code 2*$(CR = 0.1)$	28182	9.22e-09	9.22e-09	9.22e-09	9.22e-09
$F(I)3$	*pseudo-code 2*$(CR = rand)$	45545	9.27e-09	9.27e-09	9.27e-09	9.27e-09
$F(I)3$	*pseudo-code 2*$(CR = 0.9)$	1000000	8.27e+00	6.97e+00	1.11e+01	6.97e+00
$F(I)3$	$CMA - ES$	3827	4.88e+00	3.98e+00	8.95e+00	3.98e+00
$F(I)4$	*pseudo-code 2*$(CR = 0.1)$	30182	9.01e-09	9.01e-09	9.01e-09	9.01e-09
$F(I)4$	*pseudo-code 2*$(CR = rand)$	51545	2.56e-09	2.56e-09	2.56e-09	2.56e-09
$F(I)4$	*pseudo-code 2*$(CR = 0.9)$	1000000	9.94e+00	7.59e+00	1.07e+01	1.07e+01
$F(I)4$	$CMA - ES$	3691	1.35e+01	9.95e+00	2.59e+01	1.19e+01
$F(I)5$	*pseudo-code 2*$(CR = 0.1)$	3364	-1.00e-08	-1.00e-08	-1.00e-08	-1.00e-08
$F(I)5$	*pseudo-code 2*$(CR = rand)$	2091	-1.00e-08	-1.00e-08	-1.00e-08	-1.00e-08
$F(I)5$	*pseudo-code 2*$(CR = 0.9)$	1909	-1.00e-08	-1.00e-08	-1.00e-08	-1.00e-08
$F(I)5$	$CMA - ES$	199	-1.00e-08	-1.00e-08	-1.00e-08	-1.00e-08
$F(I)6$	*pseudo-code 2*$(CR = 0.1)$	1000000	9.39e-03	1.39e-03	1.81e-02	9.13e-03
$F(I)6$	*pseudo-code 2*$(CR = rand)$	63182	5.81e-09	5.81e-09	5.81e-09	5.81e-09
$F(I)6$	*pseudo-code 2*$(CR = 0.9)$	64818	9.28e-09	9.28e-09	9.28e-09	9.28e-09
$F(I)6$	$CMA - ES$	6517	-1.00e-08	-1.00e-08	-1.00e-08	-1.00e-08
$F(I)7$	*pseudo-code 2*$(CR = 0.1)$	1000000	2.99e-01	1.85e-01	4.09e-01	3.14e-01
$F(I)7$	*pseudo-code 2*$(CR = rand)$	1000000	1.48e-01	1.84e-02	3.79e-01	1.84e-02
$F(I)7$	*pseudo-code 2*$(CR = 0.9)$	48091	-2.16e-09	-2.16e-09	-2.16e-09	-2.16e-09
$F(I)7$	$CMA - ES$	1584	1.16e+00	3.60e-01	5.42e+00	3.60e-01
$F(I)8$	*pseudo-code 2*$(CR = 0.1)$	1000000	2.42e+00	1.70e+00	4.18e+00	2.35e+00
$F(I)8$	*pseudo-code 2*$(CR = rand)$	1000000	4.56e+00	4.39e+00	4.78e+00	4.39e+00
$F(I)8$	*pseudo-code 2*$(CR = 0.9)$	1000000	1.14e+00	8.54e-01	2.06e+00	8.54e-01
$F(I)8$	$CMA - ES$	7615	-1.00e-08	-1.00e-08	-1.00e-08	-1.00e-08
$F(I)9$	*pseudo-code 2*$(CR = 0.1)$	1000000	6.70e+00	6.55e+00	7.37e+00	6.55e+00
$F(I)9$	*pseudo-code 2*$(CR = rand)$	1000000	5.63e+00	5.63e+00	5.63e+00	5.63e+00
$F(I)9$	*pseudo-code 2*$(CR = 0.9)$	1000000	4.16e+00	3.98e+00	4.67e+00	4.18e+00
$F(I)9$	$CMA - ES$	7457	-1.00e-08	-1.00e-08	-1.00e-08	-1.00e-08
$F(I)10$	*pseudo-code 2*$(CR = 0.1)$	1000000	1.11e+03	5.45e+02	2.37e+03	1.09e+03
$F(I)10$	*pseudo-code 2*$(CR = rand)$	1000000	1.71e+03	1.56e+03	2.41e+03	1.56e+03
$F(I)10$	*pseudo-code 2*$(CR = 0.9)$	1000000	4.01e+03	1.24e+03	5.76e+03	5.06e+03
$F(I)10$	$CMA - ES$	7008	-1.00e-08	-1.00e-08	-1.00e-08	-1.00e-08
$F(I)11$	*pseudo-code 2*$(CR = 0.1)$	1000000	6.83e+00	6.63e+00	8.77e+00	6.63e+00
$F(I)11$	*pseudo-code 2*$(CR = rand)$	1000000	1.63e+01	1.48e+01	2.02e+01	1.48e+01
$F(I)11$	*pseudo-code 2*$(CR = 0.9)$	1000000	2.94e+01	2.15e+01	5.05e+01	2.15e+01
$F(I)11$	$CMA - ES$	6885	-1.00e-08	-1.00e-08	-1.00e-08	-1.00e-08
$F(I)12$	*pseudo-code 2*$(CR = 0.1)$	1000000	4.56e+00	2.30e+00	2.06e+01	2.30e+00
$F(I)12$	*pseudo-code 2*$(CR = rand)$	1000000	4.33e-01	1.42e-01	2.70e+00	1.42e-01
$F(I)12$	*pseudo-code 2*$(CR = 0.9)$	1000000	6.74e-02	2.54e-02	1.25e-01	4.70e-02
$F(I)12$	$CMA - ES$	15663	-1.00e-08	-1.00e-08	-1.00e-08	-1.00e-08
$F(I)13$	*pseudo-code 2*$(CR = 0.1)$	1000000	2.39e-01	1.38e-01	4.22e-01	2.58e-01
$F(I)13$	*pseudo-code 2*$(CR = rand)$	1000000	6.57e-02	8.40e-04	2.38e-01	8.40e-04
$F(I)13$	*pseudo-code 2*$(CR = 0.9)$	1000000	1.59e-02	1.63e-03	1.11e-01	1.11e-02
$F(I)13$	$CMA - ES$	4465	5.88e-05	2.25e-06	2.98e-04	9.83e-06
$F(I)14$	*pseudo-code 2*$(CR = 0.1)$	1000000	1.06e-04	1.06e-04	1.07e-04	1.06e-04
$F(I)14$	*pseudo-code 2*$(CR = rand)$	1000000	8.49e-05	8.49e-05	8.49e-05	8.49e-05
$F(I)14$	*pseudo-code 2*$(CR = 0.9)$	1000000	9.69e-05	8.82e-05	1.12e-04	8.82e-05
$F(I)14$	$CMA - ES$	10594	-1.00e-08	-1.00e-08	-1.00e-08	-1.00e-08

Table VIII: n= 10

F(I) index	Method	*EF(I)E*	error 1	error 2	error 3	error 4
$F(I)15$	*pseudo-code 2*$(CR = 0.1)$	1000000	1.37e+01	1.35e+01	1.45e+01	1.35e+01
$F(I)15$	*pseudo-code 2*$(CR = rand)$	1000000	1.20e+01	1.12e+01	1.30e+01	1.12e+01
$F(I)15$	*pseudo-code 2*$(CR = 0.9)$	1000000	1.50e+01	1.41e+01	1.93e+01	1.41e+01
$F(I)15$	$CMA-ES$	4034	1.05e+01	6.96e+00	3.08e+01	6.96e+00
$F(I)16$	*pseudo-code 2*$(CR = 0.1)$	1000000	3.55e+00	2.82e+00	5.22e+00	3.36e+00
$F(I)16$	*pseudo-code 2*$(CR = rand)$	1000000	4.20e+00	3.49e+00	6.29e+00	3.49e+00
$F(I)16$	*pseudo-code 2*$(CR = 0.9)$	1000000	3.86e+00	3.86e+00	3.86e+00	3.86e+00
$F(I)16$	$CMA-ES$	4912	1.04e-01	5.06e-02	6.43e-01	5.06e-02
$F(I)17$	*pseudo-code 2*$(CR = 0.1)$	1000000	1.05e-04	7.73e-05	2.14e-04	7.73e-05
$F(I)17$	*pseudo-code 2*$(CR = rand)$	68545	9.28e-09	9.28e-09	9.28e-09	9.28e-09
$F(I)17$	*pseudo-code 2*$(CR = 0.9)$	74000	5.82e-09	5.82e-09	5.82e-09	5.82e-09
$F(I)17$	$CMA-ES$	6296	1.26e-03	5.98e-04	1.81e-03	1.81e-03
$F(I)18$	*pseudo-code 2*$(CR = 0.1)$	1000000	3.03e-02	2.71e-02	5.36e-02	2.71e-02
$F(I)18$	*pseudo-code 2*$(CR = rand)$	1000000	4.91e-07	4.91e-07	4.91e-07	4.91e-07
$F(I)18$	*pseudo-code 2*$(CR = 0.9)$	1000000	9.90e-06	7.58e-06	1.87e-05	7.58e-06
$F(I)18$	$CMA-ES$	6106	9.16e-03	6.20e-03	1.43e-02	6.20e-03
$F(I)19$	*pseudo-code 2*$(CR = 0.1)$	1000000	9.40e-01	8.43e-01	1.24e+00	8.43e-01
$F(I)19$	*pseudo-code 2*$(CR = rand)$	1000000	9.61e-01	7.06e-01	1.42e+00	8.89e-01
$F(I)19$	*pseudo-code 2*$(CR = 0.9)$	1000000	1.27e+00	1.04e+00	1.52e+00	1.26e+00
$F(I)19$	$CMA-ES$	3917	1.45e+00	8.33e-01	3.11e+00	8.33e-01
$F(I)20$	*pseudo-code 2*$(CR = 0.1)$	57909	7.57e-09	7.57e-09	7.57e-09	7.57e-09
$F(I)20$	*pseudo-code 2*$(CR = rand)$	60000	5.66e-09	5.66e-09	5.66e-09	5.66e-09
$F(I)20$	*pseudo-code 2*$(CR = 0.9)$	1000000	6.19e-01	6.19e-01	6.19e-01	6.19e-01
$F(I)20$	$CMA-ES$	3067	1.20e+00	9.08e-01	2.03e+00	9.08e-01
$F(I)21$	*pseudo-code 2*$(CR = 0.1)$	1000000	7.12e-01	5.12e-01	1.25e+00	5.12e-01
$F(I)21$	*pseudo-code 2*$(CR = rand)$	1000000	1.25e+00	1.25e+00	1.25e+00	1.25e+00
$F(I)21$	*pseudo-code 2*$(CR = 0.9)$	1000000	1.22e+00	9.30e-01	1.25e+00	1.25e+00
$F(I)21$	$CMA-ES$	2669	1.73e+00	-1.00e-08	2.26e+00	2.00e+00
$F(I)22$	*pseudo-code 2*$(CR = 0.1)$	1000000	7.00e-01	1.87e-01	1.95e+00	1.87e-01
$F(I)22$	*pseudo-code 2*$(CR = rand)$	1000000	1.95e+00	1.95e+00	1.95e+00	1.95e+00
$F(I)22$	*pseudo-code 2*$(CR = 0.9)$	1000000	1.95e+00	1.95e+00	1.95e+00	1.95e+00
$F(I)22$	$CMA-ES$	2770	1.10e+00	-1.00e-08	5.06e+00	-1.00e-08
$F(I)23$	*pseudo-code 2*$(CR = 0.1)$	1000000	6.66e-01	4.77e-01	9.69e-01	4.77e-01
$F(I)23$	*pseudo-code 2*$(CR = rand)$	1000000	7.77e-01	7.61e-01	8.46e-01	7.61e-01
$F(I)23$	*pseudo-code 2*$(CR = 0.9)$	1000000	6.74e-01	6.74e-01	6.74e-01	6.74e-01
$F(I)23$	$CMA-ES$	3110	1.18e+00	6.41e-01	1.94e+00	6.41e-01
$F(I)24$	*pseudo-code 2*$(CR = 0.1)$	1000000	2.23e+01	2.05e+01	2.60e+01	2.05e+01
$F(I)24$	*pseudo-code 2*$(CR = rand)$	1000000	2.64e+01	2.51e+01	2.96e+01	2.65e+01
$F(I)24$	*pseudo-code 2*$(CR = 0.9)$	1000000	2.60e+01	2.39e+01	3.09e+01	2.46e+01
$F(I)24$	$CMA-ES$	3949	2.07e+01	1.22e+01	5.58e+01	1.54e+01

Table IX: n= 40

F(I) index	Method	*EF(I)E*	error 1	error 2	error 3	error 4
$F(I)1$	*pseudo-code 2*$(CR = 0.1)$	88545	9.60e-09	9.60e-09	9.60e-09	9.60e-09
$F(I)1$	*pseudo-code 2*$(CR = rand)$	1000000	4.87e-02	4.48e-02	6.36e-02	4.48e-02
$F(I)1$	*pseudo-code 2*$(CR = 0.9)$	1000000	2.21e+02	2.07e+02	2.50e+02	2.17e+02
$F(I)1$	$CMA - ES$	8643	-1.00e-08	-1.00e-08	-1.00e-08	-1.00e-08
$F(I)2$	*pseudo-code 2*$(CR = 0.1)$	1000000	4.67e-05	4.67e-05	4.67e-05	4.67e-05
$F(I)2$	*pseudo-code 2*$(CR = rand)$	1000000	6.00e+02	3.19e+02	8.23e+02	5.33e+02
$F(I)2$	*pseudo-code 2*$(CR = 0.9)$	1000000	4.06e+06	3.95e+06	5.20e+06	3.95e+06
$F(I)2$	$CMA - ES$	74733	-1.00e-08	-1.00e-08	-1.00e-08	-1.00e-08
$F(I)3$	*pseudo-code 2*$(CR = 0.1)$	1000000	3.10e+01	2.59e+01	3.39e+01	3.18e+01
$F(I)3$	*pseudo-code 2*$(CR = rand)$	1000000	9.68e+01	9.68e+01	9.68e+01	9.68e+01
$F(I)3$	*pseudo-code 2*$(CR = 0.9)$	1000000	1.37e+03	1.37e+03	1.38e+03	1.37e+03
$F(I)3$	$CMA - ES$	15792	6.18e+01	5.87e+01	8.66e+01	5.97e+01
$F(I)4$	*pseudo-code 2*$(CR = 0.1)$	1000000	3.25e+01	3.07e+01	3.46e+01	3.31e+01
$F(I)4$	*pseudo-code 2*$(CR = rand)$	1000000	1.43e+02	1.30e+02	1.82e+02	1.36e+02
$F(I)4$	*pseudo-code 2*$(CR = 0.9)$	1000000	2.01e+03	1.90e+03	2.18e+03	1.95e+03
$F(I)4$	$CMA - ES$	16160	7.96e+01	7.96e+01	7.96e+01	7.96e+01
$F(I)5$	*pseudo-code 2*$(CR = 0.1)$	8091	-1.00e-08	-1.00e-08	-1.00e-08	-1.00e-08
$F(I)5$	*pseudo-code 2*$(CR = rand)$	11636	-1.00e-08	-1.00e-08	-1.00e-08	-1.00e-08
$F(I)5$	*pseudo-code 2*$(CR = 0.9)$	5273	-1.00e-08	-1.00e-08	-1.00e-08	-1.00e-08
$F(I)5$	$CMA - ES$	719	-1.00e-08	-1.00e-08	-1.00e-08	-1.00e-08
$F(I)6$	*pseudo-code 2*$(CR = 0.1)$	1000000	1.30e+02	1.28e+02	1.53e+02	1.28e+02
$F(I)6$	*pseudo-code 2*$(CR = rand)$	1000000	1.34e+03	1.10e+03	1.92e+03	1.16e+03
$F(I)6$	*pseudo-code 2*$(CR = 0.9)$	1000000	1.41e+04	1.20e+04	1.51e+04	1.51e+04
$F(I)6$	$CMA - ES$	42486	-1.00e-08	-1.00e-08	-1.00e-08	-1.00e-08
$F(I)7$	*pseudo-code 2*$(CR = 0.1)$	1000000	7.85e+01	7.18e+01	8.33e+01	7.93e+01
$F(I)7$	*pseudo-code 2*$(CR = rand)$	1000000	2.16e+02	2.04e+02	2.30e+02	2.08e+02
$F(I)7$	*pseudo-code 2*$(CR = 0.9)$	1000000	1.16e+03	1.11e+03	1.36e+03	1.11e+03
$F(I)7$	$CMA - ES$	6842	1.28e+01	8.08e+00	1.77e+01	1.04e+01
$F(I)8$	*pseudo-code 2*$(CR = 0.1)$	1000000	6.06e+01	5.48e+01	1.19e+02	5.48e+01
$F(I)8$	*pseudo-code 2*$(CR = rand)$	1000000	1.62e+03	1.33e+03	1.85e+03	1.55e+03
$F(I)8$	*pseudo-code 2*$(CR = 0.9)$	1000000	2.40e+05	1.10e+05	3.42e+05	2.72e+05
$F(I)8$	$CMA - ES$	82472	3.62e-01	-1.00e-08	3.99e+00	-1.00e-08
$F(I)9$	*pseudo-code 2*$(CR = 0.1)$	1000000	3.77e+01	3.75e+01	3.80e+01	3.77e+01
$F(I)9$	*pseudo-code 2*$(CR = rand)$	1000000	1.91e+02	1.70e+02	2.31e+02	1.94e+02
$F(I)9$	*pseudo-code 2*$(CR = 0.9)$	1000000	1.40e+05	3.10e+04	2.03e+05	1.74e+05
$F(I)9$	$CMA - ES$	79450	-1.00e-08	-1.00e-08	-1.00e-08	-1.00e-08
$F(I)10$	*pseudo-code 2*$(CR = 0.1)$	1000000	5.18e+05	4.56e+05	6.85e+05	4.56e+05
$F(I)10$	*pseudo-code 2*$(CR = rand)$	1000000	1.89e+06	1.82e+06	2.11e+06	1.86e+06
$F(I)10$	*pseudo-code 2*$(CR = 0.9)$	1000000	4.69e+06	4.23e+06	7.80e+06	4.23e+06
$F(I)10$	$CMA - ES$	74572	-1.00e-08	-1.00e-08	-1.00e-08	-1.00e-08
$F(I)11$	*pseudo-code 2*$(CR = 0.1)$	1000000	2.74e+02	2.74e+02	2.74e+02	2.74e+02
$F(I)11$	*pseudo-code 2*$(CR = rand)$	1000000	3.77e+02	3.38e+02	4.42e+02	3.91e+02
$F(I)11$	*pseudo-code 2*$(CR = 0.9)$	1000000	4.06e+02	3.77e+02	4.81e+02	3.77e+02
$F(I)11$	$CMA - ES$	45316	-1.00e-08	-1.00e-08	-1.00e-08	-1.00e-08
$F(I)12$	*pseudo-code 2*$(CR = 0.1)$	1000000	1.97e+01	1.68e+01	3.29e+01	1.68e+01
$F(I)12$	*pseudo-code 2*$(CR = rand)$	1000000	4.17e+05	3.49e+05	6.45e+05	4.21e+05
$F(I)12$	*pseudo-code 2*$(CR = 0.9)$	1000000	3.35e+08	3.01e+08	3.93e+08	3.13e+08
$F(I)12$	$CMA - ES$	54069	-1.00e-08	-1.00e-08	-1.00e-08	-1.00e-08
$F(I)13$	*pseudo-code 2*$(CR = 0.1)$	1000000	6.97e+00	6.31e+00	9.88e+00	6.31e+00
$F(I)13$	*pseudo-code 2*$(CR = rand)$	1000000	2.67e+02	2.25e+02	2.95e+02	2.77e+02
$F(I)13$	*pseudo-code 2*$(CR = 0.9)$	1000000	2.83e+03	2.75e+03	3.01e+03	2.75e+03
$F(I)13$	$CMA - ES$	14838	9.65e-02	6.49e-04	5.08e-01	6.49e-04
$F(I)14$	*pseudo-code 2*$(CR = 0.1)$	1000000	4.30e-02	4.00e-02	4.54e-02	4.36e-02
$F(I)14$	*pseudo-code 2*$(CR = rand)$	1000000	8.55e+00	6.11e+00	1.25e+01	8.83e+00
$F(I)14$	*pseudo-code 2*$(CR = 0.9)$	1000000	6.30e+01	6.08e+01	8.51e+01	6.08e+01
$F(I)14$	$CMA - ES$	130180	-9.99e-09	-9.99e-09	-9.98e-09	-9.99e-09

Table X: n= 40

F(I) index	Method	*EF(I)E*	error 1	error 2	error 3	error 4
$F(I)15$	*pseudo-code 2*$(CR = 0.1)$	1000000	2.90e+02	2.83e+02	2.92e+02	2.92e+02
$F(I)15$	*pseudo-code 2*$(CR = rand)$	1000000	3.46e+02	3.45e+02	3.59e+02	3.45e+02
$F(I)15$	*pseudo-code 2*$(CR = 0.9)$	1000000	1.41e+03	1.33e+03	1.76e+03	1.33e+03
$F(I)15$	$CMA-ES$	18261	5.82e+01	5.67e+01	6.47e+01	5.67e+01
$F(I)16$	*pseudo-code 2*$(CR = 0.1)$	1000000	2.53e+01	2.36e+01	2.96e+01	2.36e+01
$F(I)16$	*pseudo-code 2*$(CR = rand)$	1000000	2.99e+01	2.99e+01	2.99e+01	2.99e+01
$F(I)16$	*pseudo-code 2*$(CR = 0.9)$	1000000	3.66e+01	2.88e+01	3.83e+01	3.83e+01
$F(I)16$	$CMA-ES$	27051	2.26e+00	1.95e+00	5.10e+00	2.01e+00
$F(I)17$	*pseudo-code 2*$(CR = 0.1)$	1000000	5.64e-01	4.97e-01	7.43e-01	4.97e-01
$F(I)17$	*pseudo-code 2*$(CR = rand)$	1000000	3.60e+00	3.53e+00	3.79e+00	3.53e+00
$F(I)17$	*pseudo-code 2*$(CR = 0.9)$	1000000	1.37e+01	1.27e+01	1.65e+01	1.37e+01
$F(I)17$	$CMA-ES$	26154	3.65e-02	1.61e-02	2.37e-01	1.61e-02
$F(I)18$	*pseudo-code 2*$(CR = 0.1)$	1000000	6.54e+00	5.87e+00	7.81e+00	6.74e+00
$F(I)18$	*pseudo-code 2*$(CR = rand)$	1000000	1.41e+01	1.41e+01	1.42e+01	1.41e+01
$F(I)18$	*pseudo-code 2*$(CR = 0.9)$	1000000	4.58e+01	4.37e+01	5.82e+01	4.37e+01
$F(I)18$	$CMA-ES$	27246	6.07e-01	3.35e-01	3.33e+00	3.35e-01
$F(I)19$	*pseudo-code 2*$(CR = 0.1)$	1000000	5.48e+00	4.80e+00	6.21e+00	5.99e+00
$F(I)19$	*pseudo-code 2*$(CR = rand)$	1000000	6.04e+00	5.82e+00	6.41e+00	5.86e+00
$F(I)19$	*pseudo-code 2*$(CR = 0.9)$	1000000	1.42e+01	1.41e+01	1.46e+01	1.41e+01
$F(I)19$	$CMA-ES$	8058	6.40e+00	6.22e+00	6.56e+00	6.56e+00
$F(I)20$	*pseudo-code 2*$(CR = 0.1)$	1000000	1.10e+00	1.06e+00	1.28e+00	1.06e+00
$F(I)20$	*pseudo-code 2*$(CR = rand)$	1000000	2.56e+00	2.52e+00	2.68e+00	2.52e+00
$F(I)20$	*pseudo-code 2*$(CR = 0.9)$	1000000	8.28e+04	7.65e+04	1.27e+05	7.65e+04
$F(I)20$	$CMA-ES$	12102	1.66e+00	1.40e+00	1.86e+00	1.80e+00
$F(I)21$	*pseudo-code 2*$(CR = 0.1)$	1000000	2.55e+00	2.01e+00	5.26e+00	2.07e+00
$F(I)21$	*pseudo-code 2*$(CR = rand)$	1000000	1.88e+01	1.85e+01	2.10e+01	1.85e+01
$F(I)21$	*pseudo-code 2*$(CR = 0.9)$	1000000	7.89e+01	7.87e+01	8.13e+01	7.87e+01
$F(I)21$	$CMA-ES$	8874	4.74e+00	-1.00e-08	2.98e+01	2.47e+00
$F(I)22$	*pseudo-code 2*$(CR = 0.1)$	1000000	1.96e+00	1.95e+00	1.96e+00	1.95e+00
$F(I)22$	*pseudo-code 2*$(CR = rand)$	1000000	2.01e+00	2.00e+00	2.03e+00	2.00e+00
$F(I)22$	*pseudo-code 2*$(CR = 0.9)$	1000000	7.87e+01	7.77e+01	8.37e+01	7.77e+01
$F(I)22$	$CMA-ES$	8994	8.84e+00	1.95e+00	1.46e+01	1.46e+01
$F(I)23$	*pseudo-code 2*$(CR = 0.1)$	1000000	2.67e+00	2.53e+00	3.50e+00	2.53e+00
$F(I)23$	*pseudo-code 2*$(CR = rand)$	1000000	3.59e+00	3.43e+00	3.73e+00	3.73e+00
$F(I)23$	*pseudo-code 2*$(CR = 0.9)$	1000000	3.46e+00	3.18e+00	3.96e+00	3.18e+00
$F(I)23$	$CMA-ES$	8458	3.24e+00	3.24e+00	3.24e+00	3.24e+00
$F(I)24$	*pseudo-code 2*$(CR = 0.1)$	1000000	2.94e+02	2.86e+02	3.24e+02	2.86e+02
$F(I)24$	*pseudo-code 2*$(CR = rand)$	1000000	3.59e+02	3.51e+02	3.91e+02	3.51e+02
$F(I)24$	*pseudo-code 2*$(CR = 0.9)$	1000000	1.07e+03	1.03e+03	1.12e+03	1.09e+03
$F(I)24$	$CMA-ES$	9568	1.02e+02	5.43e+01	3.29e+02	5.43e+01
